# Specific lineage transition of tumor-associated macrophages elicits immune evasion of ascitic tumor cells in gastric cancer with peritoneal metastasis

**DOI:** 10.1007/s10120-024-01486-6

**Published:** 2024-03-09

**Authors:** Yilin Li, Lei Jiang, Yang Chen, Yanyan Li, Jiajia Yuan, Jialin Lu, Zizhen Zhang, Shengde Liu, Xujiao Feng, Jiaxin Xiong, Yan Jiang, Xiaotian Zhang, Jian Li, Lin Shen

**Affiliations:** 1 State Key Laboratory of Holistic Integrative Management of Gastrointestinal Cancers, Beijing Key Laboratory of Carcinogenesis and Translational Research, Department of Gastrointestinal Oncology, Peking University Cancer Hospital & Institute, Beijing, 100142 China; 2 Singleron Biotechnologies, Nanjing, China

**Keywords:** Tumor-associated macrophages, Immune evasion, Ascitic tumor, Gastric cancer, Peritoneal metastasis

## Abstract

**Background:**

Gastric cancer with peritoneal metastasis (PM-GC), recognized as one of the deadliest cancers. However, whether and how the tumor cell-extrinsic tumor microenvironment (TME) is involved in the therapeutic failure remains unknown. Thus, this study systematically assessed the immunosuppressive tumor microenvironment in ascites from patients with PM-GC, and its contribution to dissemination and immune evasion of ascites-disseminated tumor cells (aDTCs).

**Methods:**

Sixty-three ascites and 43 peripheral blood (PB) samples from 51 patients with PM-GC were included in this study. aDTCs in ascites and circulating tumor cells (CTCs) in paired PB were immunophenotypically profiled. Using single-cell RNA transcriptional sequencing (scRNA-seq), crosstalk between aDTCs and the TME features of ascites was inspected. Further studies on the mechanism underlying aDTCs-immune cells crosstalk were performed on in vitro cultured aDTCs.

**Results:**

Immune cells in ascites interact with aDTCs, prompting their immune evasion. Specifically, we found that the tumor-associated macrophages (TAMs) in ascites underwent a continuum lineage transition from cathepsin^*high*^ (CTS^*high*^) to complement 1q^*high*^ (C1Q^*high*^) TAM. CTS^*high*^ TAM initially attracted the metastatic tumor cells to ascites, thereafter, transitioning terminally to C1Q^*high*^ TAM to trigger overproliferation and immune escape of aDTCs. Mechanistically, we demonstrated that C1Q^*high*^ TAMs significantly enhanced the expression of PD-L1 and NECTIN2 on aDTCs, which was driven by the activation of the C1q-mediated complement pathway.

**Conclusions:**

For the first time, we identified an immunosuppressive macrophage transition from CTS^*high*^ to C1Q^*high*^ TAM in ascites from patients with PM-GC. This may contribute to developing potential TAM-targeted immunotherapies for PM-GC.

**Graphical Abstract:**

**Schematic of the immune TME of ascites and the crosstalk with aDTCs in patients with PM-GC**. In ascites with TAM-dominant TME, the ascitic TAMs undergo CTS-to-C1Q transition to support multiple phases of aDTC dissemination, including aDTC homing, proliferation, immune escape, and therapeutic resistance. While in ascites with T-cell-dominant TME, enriched T cells do not imply “immune-hot” TME. Infiltrated CD8^+^ T cells are GZMK^+^ precursor-exhausted cells that have lost their capacity to kill tumor cells. (Abbreviations: *aDTC* ascites-disseminated tumor cells, *CTS* cathepsin, *TAM* Tumor-associated macrophages, *TME* tumor microenvironment)

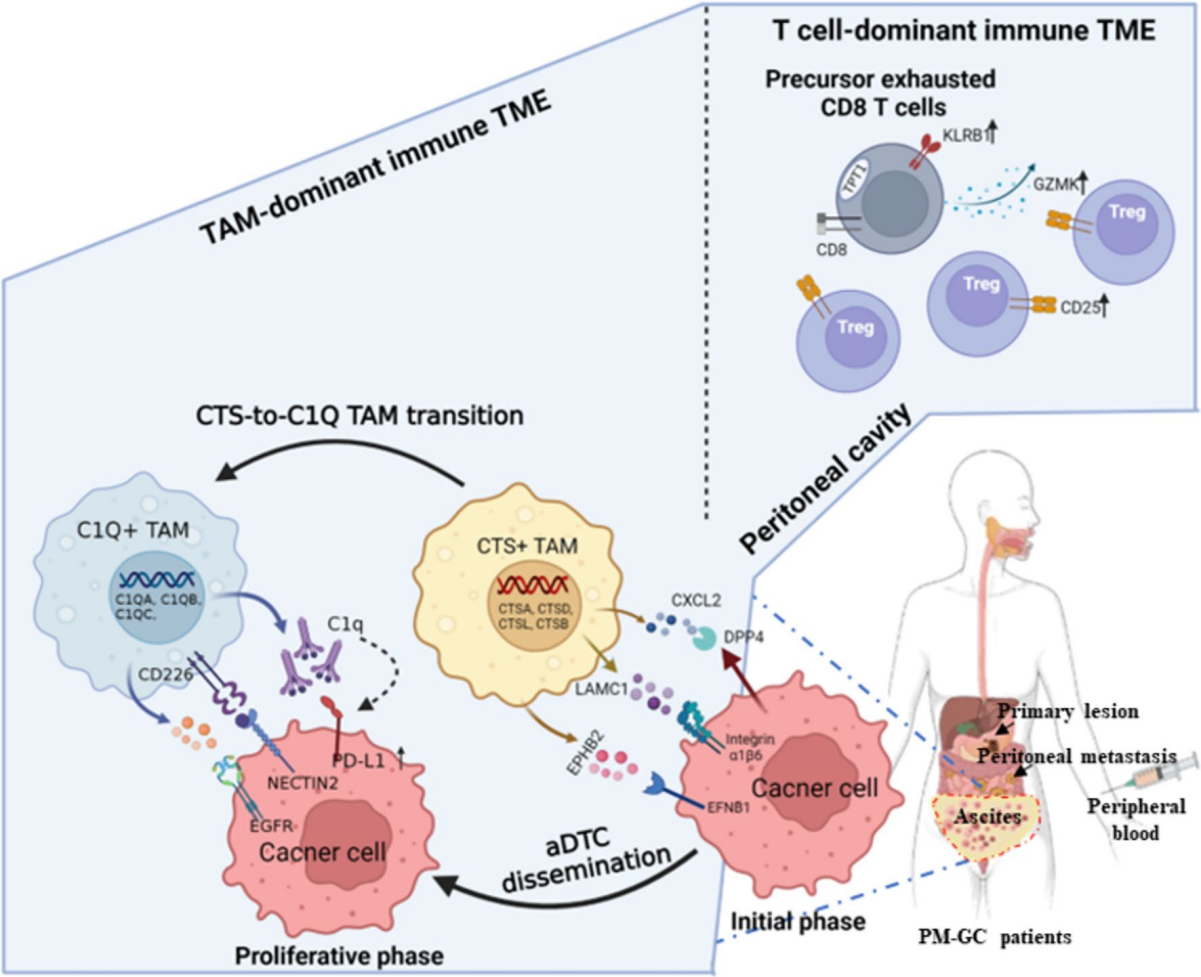

**Supplementary Information:**

The online version contains supplementary material available at 10.1007/s10120-024-01486-6.

## Introduction

Peritoneal metastasis (PM), a fatal type of fatal metastasis, can develop in over 15% of patients with gastric cancer (GC) [1]. However, chemotherapies and targeted therapies have failed to prevent GC with peritoneal carcinomatosis (PM-GC) onset [2, 3]. Although recent immunotherapies have achieved excellent success in other cancers, including non-small-cell lung cancer [4], melanoma [5], and microsatellite instability-high (MSI-H) colorectal cancer [6], their effectiveness is limited in patients with PM-GC [7], indicating the high immunosuppressive characteristics of PM-GC.

PM-GC presents clinical therapeutic resistance to the increased cell-intrinsic aggressiveness of metastatic tumor cells [8–11]. Tumor cells disseminating into malignant ascites, i.e., ascites-disseminated tumor cells (aDTCs), are associated with high epithelial-to-mesenchymal plasticity [8, 9], continuous transcriptional activation, and transforming growth factor-β (TGF-β) pathway activation [8], possibly upregulating immune checkpoints and consequently inducing aDTC immune evasion from immune surveillance and immunotherapeutic pressure [8]. Moreover, evidence from other cancers suggests that the tumor microenvironment (TME), especially immune cells in the TME, can interplay with tumor cells, immunoediting their malignancy and resistance to therapeutic pressures [12]. Furthermore, tumor-associated macrophages (TAMs) are “pro-tumor” immune cells in high numbers in the TME [13] and are crucial in maintaining cancer stem cell (CSC) stemness; for instance, in breast cancer, TAMs can secrete regulatory mediators, including transforming growth factor-β1 (TGF-β1), to improve CSC epithelial-to-mesenchymal transition, prompting their tolerance to treatment pressures [14]. Additionally, juxtacrine secreted from glioblastoma TAMs activates PTPRZ1 signaling in glioblastoma CSCs, fostering tumor growth [15]. However, the TME composition in peritoneally metastatic niches in PM-GC remains poorly understood [16]. Furthermore, how immunosuppressive TME components, including TAMs, affect tumor-cell immune evasion and immunotherapeutic resistance in PM-GC remains unclear.

Thus, this systematic study assessed immune cells in the TME and their interaction with aDTCs based on three independent PM-GC cohorts containing 63 ascites from 51 patients with GC and provided a new perspective for immunotherapy in PM-GC by targeting the TAM transition and its crosstalk.

## Materials and methods

### Specimen collection

This study was conducted at Peking University Cancer Hospital, Bei Jing, China from July 2019 to June 2021. The study used three independent cohorts with 63 ascites and 43 peripheral blood samples from 51 patients with PM-GC (Supplementary Fig. S1). Detailed information regarding the three cohorts, subtraction enrichment and immunostaining-fluorescence in situ hybridization (SE-iFISH), single-cell RNA sequencing (scRNA-seq) test, and scRNA-seq validation, is shown in Supplementary Fig. S1 and supplementary materials and methods.

### aDTCs or circulating tumor cells (CTCs) enrichment by SE-iFISH

The ascites and matched paired peripheral blood (PB) samples in the SE-iFISH cohort were subjected to aDTC or CTC detection using the SE-iFISH platform. The experiment was performed according to the manufacturer’s protocol (Cytelligen, San Diego, CA, USA) with minor modifications. Briefly, for aDTCs or CTCs detection, ascitic fluid or peripheral blood samples were collected in tubes containing ACD anticoagulant (Becton Dickinson, Franklin Lakes, NJ, USA). The collected samples were then centrifuged at 350–400 g for 15 min. The sedimented cells were mixed with hCTC buffer, loaded on the non-hematopoietic cell separation, and centrifuged at 350 g for 6 min. The entire solution above the red blood cells (RBCs) layer was then collected and incubated with immuno-magnetic beads conjugated with a cocktail of anti-leukocyte monoclonal antibodies (Cytelligen, San Diego, CA, USA) for 20 min. White blood cells (WBCs) bound to immuno-beads were removed using a magnetic separator. The solution free of magnetic beads was collected, washed using phosphate-buffered saline (PBS), and spun twice to remove unspecific binding mAbs. Thereafter, the sedimented cells were processed for iFISH.

The dried monolayer cells on the coated aDTC/CTC slides were hybridized with CEP8 Spectrum Orange (Vysis, Abbott Laboratories, Chicago, IL, USA). Samples were subsequently incubated with anti-CD45 mAb conjugated with Alexa Fluor (AF) 594 (Cytelligen, San Diego, CA, USA). After washing, the samples were mounted using mounting media and scanned and analyzed with the automated Metafer-ifluorescence in situ hybridization (FISH)® 3D scanning (Carl Zeiss, Oberkochen, Germany) and image analysis system (MetaSystems, Altlus-sheim, Germany), FISH was performed using a centromere probe 8 (CEP8, Vysisy and Abbott, Abbott Park, IL, USA). In SE-iFISH platform, aneuploid Chr8 was employed as a marker for tumor cell confirmation due to the heterogenous expressions of epithelial and mesenchymal markers on DTCs (Supplementary Fig. S2). Overall, the aDTCs/CTCs with > disomy 8 were defined as aneuploids. Moreover, aDTCs/CTCs were defined as DAPI^+^ and CD45^−^ with aneuploid Chr8 [17, 18].

### Single-cell RNA sequencing

For scRNA-seq, single-cell suspensions in PBS (HyClone) with a concentration of 2 × 10^5^ cells/mL were firstly prepared and then loaded onto microfluidic devices using the Singleron Matrix® Single Cell Processing System, and scRNA-seq libraries were constructed according to the GEXSCOPE® protocol using the GEXSCOPE® Single-Cell RNA Library Kit (Singleron Biotechnologies Köln, Germany). The individual libraries were diluted to 4 nM and pooled for sequencing on the Illumina Novaseq 6000 with 150-bp paired-end reads.

### Primary analysis of raw read data

Raw reads obtained from the scRNA-seq were processed to generate gene expression matrixes using the CeleScope v1.3.0 (https://github.com/singleron-RD/CeleScope), an internal pipeline. Briefly, the raw reads were processed with the CeleScope to remove low-quality reads. The raw reads were first processed with fastQC v0.11.4 [19] and fastp (https://github.com/OpenGene/fastp) to remove low-quality reads and trim the poly-A tail and adapter sequences. The cell barcode and unique molecular identifier (UMI) were extracted. Thereafter, we used STAR v2.5.3a (https://github.com/alexdobin/STAR) to map the reads to the reference genome GRCh38 (ensemble version 92 annotation). The UMI and gene counts of each cell were obtained using Counts v1.6.2 (https://subread.sourceforge.net/featureCounts.html), and these were used to generate the expression matrix files for subsequent analysis.

### Quality control, dimension reduction, and clustering

Scanpy v1.8.2 (https://scanpy.readthedocs.io/en/stable/) was used for quality control, dimensionality reduction, and clustering under Python 3.7. Finally, the cell clusters were visualized using the Uniform Manifold Approximation and Projection (UMAP). The details please see the supplementary materials and methods.

### Differentially expressed gene (DEG) analysis

To identify DEGs, we used the Seurat Find Markers function based on the Wilcox likelihood-ratio test with default parameters, and we selected genes expressed in > 10% of cells in a cluster and with an average log (Fold Change) value > 0.25 in DEGs. For the cell type annotation of each cluster, we combined the expression of canonical markers found in DEGs based on the Scanpy literature and displayed the expression of markers of each cell type with heatmaps/dot plots/violin plots that were generated with the Seurat DoHeatmap/DotPlot/Vlnplot function. The mean expression of each cluster/sample was calculated using the Seurat Average Expression function. Doublet cells were identified as expression markers for different cell types and removed manually.

### Cell type annotation and cell subtype identification

The cell type identity of each cluster was determined with the expression of the canonical markers found in the DEGs using the SynEcoSys database (Supplementary Table S1). In the scRNA-seq test cohort, we further subclustered the epithelial cells, T cells, MPs, and macrophages at resolutions 0.8, 1.0, 1.2, and 0.2, respectively. For the scRNA-seq validation cohort, we subclustered MPs and macrophages at resolutions 1.2 and 0.5, respectively.

### scRNA-seq-based copy number variation (CNV) detection

The InferCNV package was used to detect CNVs in subpopulations of malignant epithelial cells (https://jlaffy.github.io/infercna). Non-malignant cells (mononuclear phagocytes [MPs], T cells, and B cells) were used as baselines to estimate the CNVs of malignant cells. The genes expressed in over 20 cells were sorted according to their loci on each chromosome. The relative expression values were centered to one using a 1.5 standard deviation from the residual-normalized expression values as the ceiling. A slide window size of 101 genes was used to smooth the relative expression on each chromosome to remove the effect of the gene-specific expression.

### Trajectory analysis

Trajectory analysis was performed to track cell transition statuses. Cell data were reprocessed to remove low-UMI count genes or low-quality cells and were renormalized for the library size using the R package Monocle. After completing the quality control process, dimensional reduction and trajectory construction were conducted. The cells were placed on a pseudotime trajectory using the order obtained from the cell function. The pseudotime trajectory was inferred from the root cells that comprised the annotated macrophages. The differentiation trajectory of the macrophage subpopulations was reconstructed using Monocle 2 (https://github.Scylardor/Monocle2). Highly variable genes were used to sort cells according to spatial‐temporal differentiation. Moreover, we used the DDR Tree (Discriminative Dimensionality Reduction via learning a Tree) (https://github.com/cole-trapnel-lab/DDRTree) to perform FVF and dimension reduction. Finally, the trajectory was visualized using the plot cell trajectory function.

#### Cell–cell interaction analysis

Cell–cell interaction analysis was performed using CellPhoneDB v2.1.0 (https://github.com/Teichlab/cellphonedb) based on the known receptor–ligand interactions between the two cell types/subtypes. The cluster labels of all cells were permuted randomly 1000 times to calculate the null distribution of the average ligand-receptor expression levels of the interacting clusters. The individual ligand or receptor expression was accorded a threshold value with the cutoff value based on the average log gene expression distribution for all genes across all cell types. The significant cell–cell interactions were defined with a *P*-value of < 0.05 and an average log expression of > 0.1, which were visualized with the Circlize v0.4.10 R package (https://jokergoo.github.io/2020/06/14/changes-in-circlize-0.4.10/).

#### In vitro culturing of ascitic-derived primary tumor cells

Ascitic fluid was collected in sterile containers and used immediately to establish the primary cultures. The details please see the supplementary materials and methods.

#### Construction of TurboID biotin labeling system and interaction proteomic analysis

The TurboID biotin labeling system [20, 21] was used to biotinylate cytokines in ascites supernatant that interact with in vitro cultured ascites tumor cells. Briefly, ascites tumor cells were firstly transfected with lentivirus expressing TurboID (detailed procedures in Supplementary Materials and Methods). When incubating the TurboID-expressed ascitic tumor cells with their matched ascitic supernatants, proteins interacting with ascitic tumor cells in supernatants were near TurboID and were thereby biotinylated. Accordingly, streptavidin magnetic beads were utilized to pull down the biotinylated proteins. Moreover, all enriched biotinylated proteins were analyzed by liquid chromatography-mass spectrometry/mass spectrometry (LC–MS/MS) for further proteomic analysis. Procedures for LC–MS/MS detection and data analysis are shown in supplementary materials and methods.

#### Flow cytometric analysis

All antibodies used in flow cytometric analysis and the manufacturers’ information are listed in supplementary materials and methods.

#### Enzyme-linked immunosorbent assay (ELISA)

All the ELISA kits used are listed in supplementary materials and methods.

#### Western blot

All antibodies used in western blotting are listed in supplementary materials and methods.

#### Statistical analysis

All statistical analyses were performed using SPSS 21.0 (IBM Corp., NY, USA) or GraphPad Prism V8 (GraphPad Software, San Diego, CA, USA). For scRNA-seq data analysis, cell distribution comparisons between the two groups were performed using unpaired two-tailed Wilcoxon rank-sum tests. The comparison of the gene expression or gene signature between the two groups of cells was performed using the unpaired two-tailed Student’s t-test. Paired two-tailed Wilcoxon rank-sum tests were used to compare cell distribution between Group 1 and Group 2 and between ascites and matched PB samples. All statistical analyses and visualizations were conducted in R. The statistical tests used in the figures are indicated in the figure legends, and statistical significance was set at *P*-values < 0.05.

## Results

### Tumor cell-extrinsic dysfunctions also regulate aDTC immune evasion

**Fig. 1:**
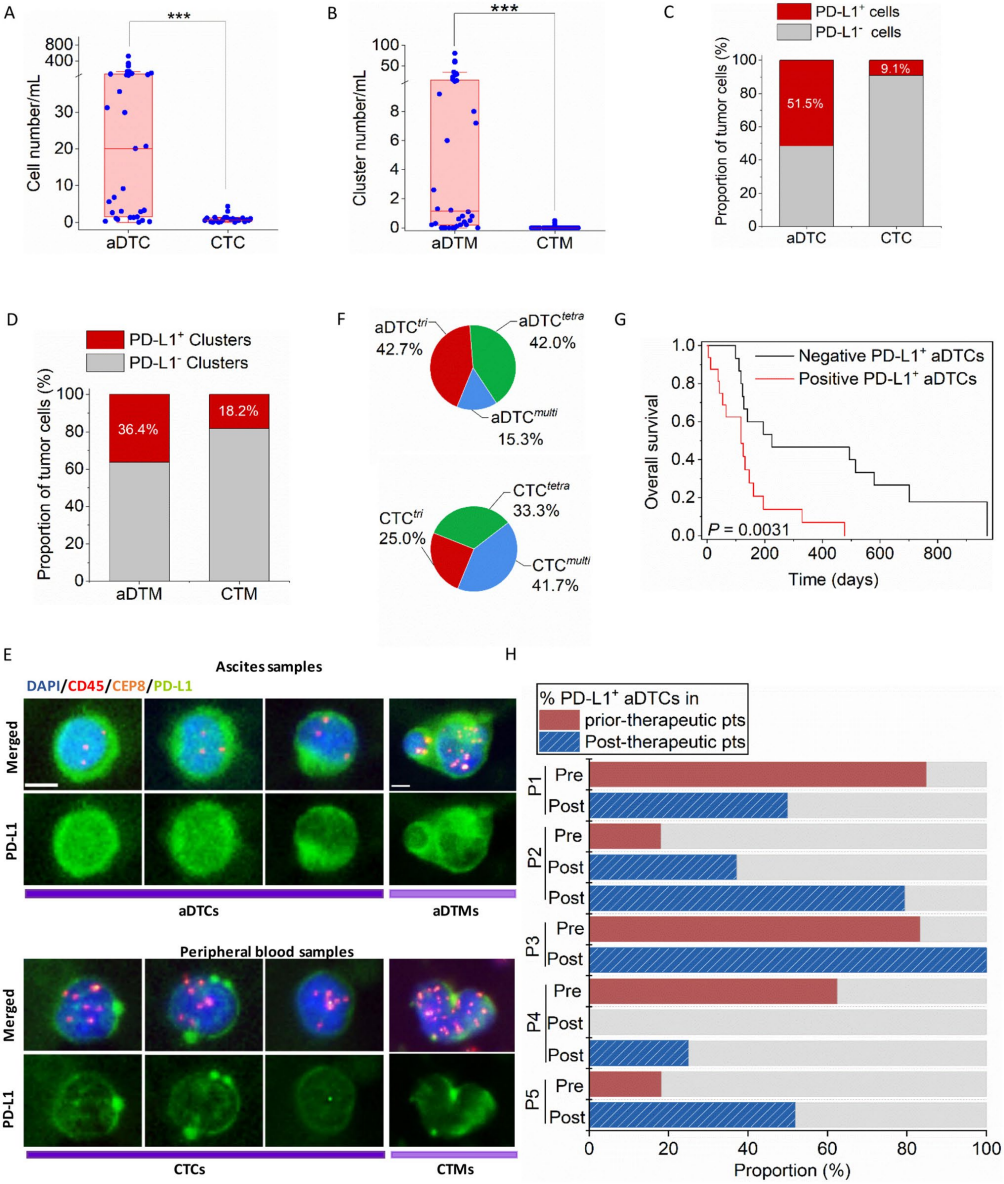
**Upregulation of PD-L1 on aDTCs mediates immune escape and deteriorating prognosis in PM-GC**. **a** and **b** Quantitative comparison of aDTCs (**a**) and aDTMs (**b**) in ascites (*N* = 33) with CTCs (**a**) or CTMs (**b**) in the paired peripheral blood samples (*N* = 33). Data was statistically analyzed using the non-parametric Mann–Whitney test. ^***^P < 0.001. **c** and **d** Comparison of proportions of PD-L1 + aDTCs (**c**) or aDTMs (**d**) within total aDTCs or aDTMs from ascites samples (*N* = 33) to PD-L1 + CTCs (**c**) or CTMs (**d**) within total CTCs or CTMs from paired peripheral blood samples (*N* = 33). **e** Representative immunofluorescent images showing PD-L1 expression on aDTCs or aDTM and CTCs or CTM from the same patient using SE-iFISH (bar = 5 µm). **f** Proportions of chromosome 8 (chr 8) triploidy, tetraploidy, and multiploidy in total detected PD-L1^+^ aDTCs from ascites samples (*N* = 33, upper pie chart) or PD-L1^+^ CTCs from paired peripheral blood samples (*N* = 33, lower pie chart). **g** Kaplan–Meier plots illustrating the overall survival of patients with negative PD-L1^+^ aDTCs and that of those with positive PD-L1^+^ aDTCs. **h** Proportional variations of PD-L1^+^ aDTCs following treatments. (Abbreviations: *aDTC* ascites-disseminated tumor cells, *CTS* cathepsin, *CTM* circulating tumor microemboli; aDTC; CTC; aDTM; CTM; aDTC^tri^ and CTC^tri^ are chr 8 triploid aDTC and CTC; aDTC^tetra^ and CTC^tetra^ are chr 8 tetraploid aDTC and CTC; aDTC^multi^ and CTC^multi^ are chr 8 multiploid aDTCs and CTCs.)

Previous bulk transcriptional sequencing (RNA-seq) identified the overexpressed immune checkpoints TIM-3 and VISTA in ascitic cells of patients with PM-GC, indicating ascitic cells’ high immune escape ability [9]. To re-confirm this, aDTCs from ascites and CTCs from paired PB samples of 33 treatment-naïve patients with PM-GC were enriched via the previously established SE-iFISH platform (SE-iFISH cohort, Supplementary Fig. S1 and Table S2). Additionally, a pairwise comparison of the expression of PD-L1, another immune checkpoint marker mediating immune escape [22, 23], was conducted on enriched aDTCs or CTCs. The enumeration total and expression levels of PD-L1^+^ on aDTCs or ascites-disseminated microemboli (aDTMs), also known as ascites-disseminated tumor cell clusters aggregated with two or more aDTCs, were all higher than those of CTCs or circulating tumor microemboli (CTMs) [24] (Fig. 1a–d). In individual patients, the expression level of PD-L1 on aDTCs and aDTMs were augmented compared with that on paired CTCs and CTMs (Fig. 1e).

Besides enumeration and expression levels, the karyotypic characteristics of PD-L1^+^ on aDTCs and CTCs are completely distinct. PD-L1^+^ CTCs are predominantly multiploid chromosome 8 (chr8) (CTC^*multi*^), whereas PD-L1^+^ aDTCs are mainly triploid chr8 (aDTC^*tri*^), indicating an elevated therapeutic tolerance of PD-L1^+^ aDTCs (Fig. 1f) [25]. Moreover, the prognostic analysis of the SE-iFISH cohort (Cohort-1) confirmed shorter overall survival (OS) of patients with positive pre-therapeutic PD-L1^+^ aDTCs (PD-L1^+^ aDTCs ≥ 1 cell/6 mL, Fig. 1g). These results are in line with previous findings and further demonstrated that the overexpressed PD-L1 on aDTCs is involved in aDTC immune evasion.

Current studies on PD-L1^+^ tumors suggest a paradoxical role of the PD-L1 expression on the PD-1/PD-L1 blockade efficiency [26, 27]. Although higher pre-therapeutic PD-L1 levels in primary tumors are considered an indicator of increased sensitivity to anti-PD-1/PD-L1 therapy, the re-acquisition or uncontrollable PD-L1 expression on either tumor or immune cells is associated with therapeutic resistance to PD-1/PD-L1 blockades [27]. Our results indicated that disruption of tumor-cell intrinsic aggressiveness by systematic chemotherapies could not abolish overexpressed PD-L1 on aDTCs, thereby demonstrating that PD-L1^+^ aDTCs were still detectable in most post-therapy ascites in 5 of 33 enrolled patients (Fig. 1h). This implies that re-acquisition or uncontrolled PD-L1 overexpression may be regulated by tumor cell-intrinsic mechanisms and tumor cell-extrinsic mechanisms. Therefore, inhibiting only tumor-cell intrinsic targets cannot completely curb aDTC immune escape.

### MPs dominate the TME of aDTC-rich ascites

**Fig. 2:**
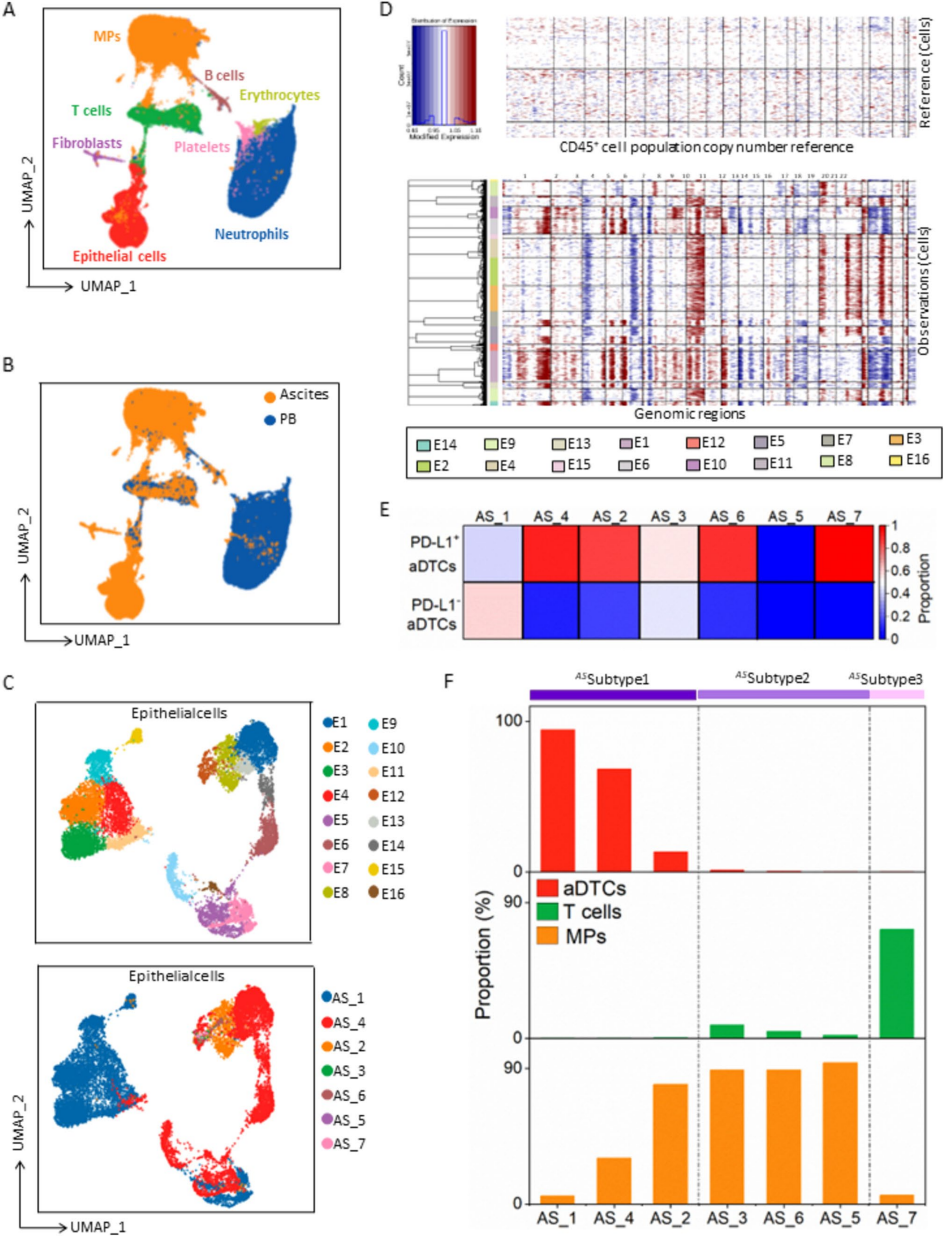
**scRNA-seq of seven treatment-naïve ascites and paired peripheral blood samples from the scRNA-seq test cohort**. **a** and **b** UMAP representation of all annotated cell clusters from seven ascites and paired peripheral blood samples. Cell clusters are colored according to cell type (**a**) or sample origin (**b**). **c** Unsupervised clustering of epithelial cells from ascites. The upper UMAP is colored according to epithelial cell clusters, and the lower UMAP is colored according to different ascites samples. **d** Copy number variations (CNV) in epithelial cell clusters. Columns represent epithelial clusters, and rows represent chromosomal regions. The CNVs of CD45^+^ cells serve as references. **e** The proportion of PD-L1^+^ aDTCs in total aDTCs detected in seven treatment-naïve ascites from the scRNA-seq test cohort using SE-iFISH. Each column represents a unique ascites sample, and the two rows represent the proportion of PD-L1^+^ aDTCs and PD-L1^−^ aDTCs, respectively. **f** Histograms displaying the proportions of aDTCs, T cells, and mononuclear phagocytes (MPs) in seven ascites samples from the scRNA-seq test cohort. The absolute number of aDTCs, T cells, and MPs is shown in **Supplemental Fig. 2C**. (Abbreviations: *aDTC* ascites-disseminated tumor cells, ^*AS*^*Subtype1* aDTC-rich ascites, ^*AS*^*Subtype2* MP-dominant ascites, ^*AS*^*Subtype3* T-dominant ascites, *UMAP* Uniform Manifold Approximation and Projection)

To comprehensively understand tumor-cell extrinsic mechanisms underlying aDTC immune evasion, we cataloged the cell components of ascites and paired PB samples collected from seven treatment-naïve patients with PM-GC (scRNA-seq test cohort, Supplementary Fig. S1 and Supplementary Table S3) by leveraging single-cell transcriptional sequencing (scRNA-seq). Overall, 144,522 cells from all 14 ascites samples and matched PB samples, which passed multiple quality controls, were further clustered using Seurat. Overall, eight cell clusters, including MPs [28], epithelial cells [29], T cells [30], B cells [31], platelets [32], fibroblasts [33], erythrocytes [32], and neutrophils [34], were annotated using canonical markers (Fig. 2a, Supplementary Fig. S3A, and Supplementary Table S1). Among these, epithelial cells and MPs were mainly from ascites samples, while T and B cells were from both ascites and PB samples (Fig. 2b, Supplementary Fig. S3B-C).

Since aDTC presence is a prognosticator of an inferior OS in PM-GC (Supplementary Fig. S3D), we first inspected the malignant subsets in epithelial cells. In the predominantly enriched samples AS_1, AS_4, and AS_2, 16 epithelial cell clusters were identified (Fig. 2c and Supplementary Fig. S3C). Consistent with previous findings [10], the epithelial cells in the ascites samples from PM-GC patients were highly heterogenous, and each ascites sample comprised specific epithelial cell clusters. For instance, the epithelial cell clusters E2, E3, E4, and E9 were only found in sample AS_1. Conversely, the clusters E6, E10, and E14 belonged exclusively to sample AS_4 (Fig. 2c and Supplementary Fig. S3E). High diversity of CNVs was identified in the individual clusters, further demonstrating malignancy in all 16 epithelial cell clusters; accordingly, we referred to all epithelial cell clusters as aDTC clusters (Fig. 2d). The 17q copy number gain, which is considered a unique event in tumor cells from the stomach [10], was identified in most aDTC clusters in our study, confirming the stomach tumor-specific origin of all detected aDTC clusters (Fig. 2d).

Regarding PD-L1 expression, we failed to recognize the transcriptional PD-L1 elevation in aDTC clusters, probably owing to the dropouts in scRNA-seq. However, PD-L1 protein overexpression on aDTCs was validated by SE-iFISH, as demonstrated by > 50% of aDTCs being detected as PD-L1-positive in five out of seven ascites samples (Fig. 2e).

We hypothesized that the upregulated PD-L1 on aDTCs and its immune escape mediation is modulated by immunosuppressive interactions between the cell-extrinsic immune TME and aDTCs. To demonstrate this, the immune components, especially in the aDTC-rich ascites samples (samples AS_1, AS_4, and AS_2: referred to as ^*AS*^Subtype 1), were analyzed. MPs, rather than T cells, were overwhelmingly infiltrated in the TME of the aDTC-rich ascites samples (Fig. 2f and Supplementary Fig. S3C). Moreover, aDTC quantification in aDTC-rich ascites showed an inverse correlation with the presence of MPs, implying robust communications between aDTCs and MPs (Fig. 2f).

In aDTC-deficient ascites, MPs and T cells may exist exclusively in the TME of the ascitic fluid, which further shapes the two heterogenous TME subtypes of ascites: ^*AS*^Subtype 2 and ^*AS*^Subtype 3. In ^*AS*^Subtype 2 ascites, the TME is MP-dominant, with < 10% T cells being infiltrated (sample AS_3, AS_6, and AS_5, Fig. 2f). In ^*AS*^Subtype 3 ascites, a high presence of T cells in the TME impedes MP infiltration (sample AS_7, Fig. 2f). These results indicated that MPs and T cells might independently modulate the development of ascites in patients with PM-GC.

### Ascitic TAMs form a continuum transitioning from cathepsin (CTS)^***high***^ to complement 1q (C1Q) ^***high***^

**Fig. 3:**
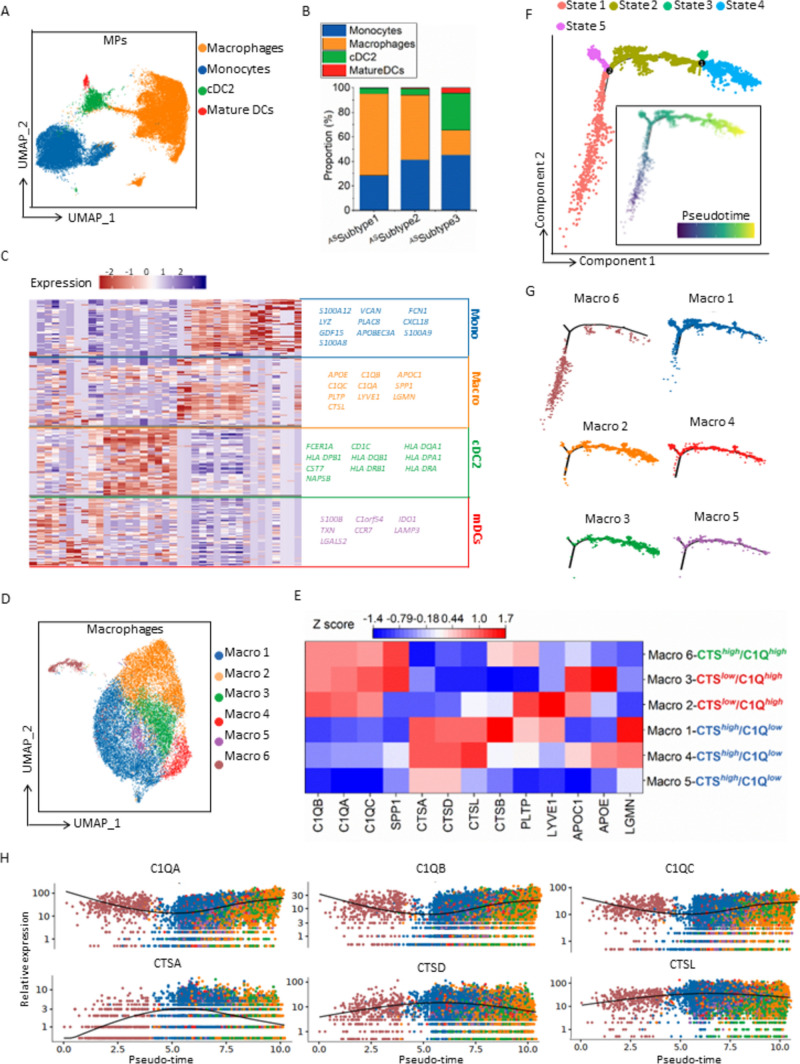
**scRNA-seq analysis of the ascitic TAMs of seven treatment-naïve ascites from the scRNA-seq test cohort**. **a** UMAP representation of annotated subpopulations of MPs. Different colors are correlated to different cell types of MPs. **b** Proportions of individual MP subpopulations in three ascitic subtypes. **c** Heatmap showing the top 10 differentially expressed genes (DEGs) in each MP subpopulation. Columns represent normalized expressions of DEGs, and each row represents a cell. Mono, monocytes; Macro, macrophage; cDC2, type 2 conventional dendritic cell; mDCs, mature dendritic cells. **d** The UMAP of the unsupervised clustering of macrophages. Each color represents a unique macrophage cluster. **e** Average expressions of DEGs in macrophages identified from panel c in the individual macrophage cluster. Each column represents the average gene expression normalized by Z score normalization, and each row represents a unique macrophage cluster. **f** Trajectory analysis of the differentiation of TAMs. Cells on the trajectories are colored according to their states (outer graph) or pseudotime (inner graph). **g** Respective projection of C1Q^*high*^/CTS^*high*^ (Macro 6), C1Q^*high*^ (Macro 2 and 3), and CTS^*high*^ (Macro 1, 4, and 5) TAM lineages onto the trajectory of TAM differentiation. **h** Variation of the expressions of C1Q or CTS genes following pseudotime. Each dot in the graphs represents a unique cell. Color coding is explained in (**g**). (Abbreviations: *MPs* mononuclear phagocytes, *TAM* Tumor-associated macrophages, *UMAP* Uniform Manifold Approximation and Projection

Physiologically, a macrophage plays a critical role in maintaining immune homeostasis in the peritoneal cavity [1]. This raises the question of whether TAMs are hub MPs involved in immunosuppressive communication with aDTCs in PM-GC ascites. Moreover, macrophages account for the highest MP proportion, especially in the aDTC-rich ^*AS*^Subtype 1 ascites (Fig. 3a, b and Supplementary Fig. S4A). Additionally, the upregulated DEGs identified in macrophages, including APOE, C1QB, APOC1, C1QC, C1QA, SPP1, LYVE1, LGMN, and cathepsin L (CTSL), are all anti-inflammatory, further conferring their immunosuppressive function; accordingly, we referred to all annotated macrophages as TAMs (Fig. 3c). To disclose the specific function of the individual TAM lineage clustered by unsupervised learning (Fig. 3d and Supplementary Fig. S4B), we initially examined canonical M1 and M2 signatures in each TAM cluster [16]. Nevertheless, all TAM clusters failed to fall into the M1/M2 dichotomy (Supplementary Fig. S4C and Supplementary Table S4). However, when comparing DEGs of TAMs from the MP clustering (Fig. 3c) in the individual TAM lineage, we noted the presence of transitionally varied expression patterns of the C1Q-associated genes C1QA, C1QB, and C1QC and CTS-associated genes CTSA, CTSD, CTSL, which can confer three TAM lineages perfectly: CTS^*high*^/C1Q^*high*^ (Macro 6), CTS^*low*^/C1Q^*high*^ (Macro 3 and Macro 2), and CTS^*high*^/C1Q^*low*^ (Macro 1, Macro 4, and Macro 5) (Fig. 3e). These indicated that the expressional variation of CTS and C1Q genes on ascitic TAMs are a continuum instead of a dichotomic polarization.

Therefore, we investigated how this continuum of gene expression varied following TAM lineage differentiation. The trajectory of TAM lineages analyzed using Monocle 2 is shown in Fig. 3f. The five developmental states of TAM lineages were determined. The differentiation of ascitic TAMs commenced from the CTS^*high*^/C1Q^*high*^ Macro 6, evolving to CTS^*high*^ TAM lineages (Macro 1, 4, and 5) at intermediate states (Fig. 3g and Supplementary Fig. S4D). Distinct from the CTS^*high*^ TAM lineages that exist in all states thereafter, the C1Q^*high*^ TAM lineages (Macro 2, and 3) were only developed in the terminal state (Fig. 3g and Supplementary Fig. S4D). Further insight into the expression of C1Q and CTS genes following pseudotime confirmed that C1Q gene levels were only elevated at the terminal pseudotime. This was contrary to the CTS genes, which showed a terminal decrease after an increase at the intermediate pseudotime (Fig. 3h).

Altogether, the obtained results support the view that ascitic TAMs undergo a lineage transition from the CTS^*high*^ to C1Q^*high*^ subtype (CTS-to-C1Q transition) and that the CTS^*high*^ lineages are TAM precursors, which differentiate terminally toward the C1Q^*high*^ lineages.

### TAM transition from CTS^***high***^ to C1Q^***high***^ modulates different aDTC dissemination phases

**Fig. 4:**
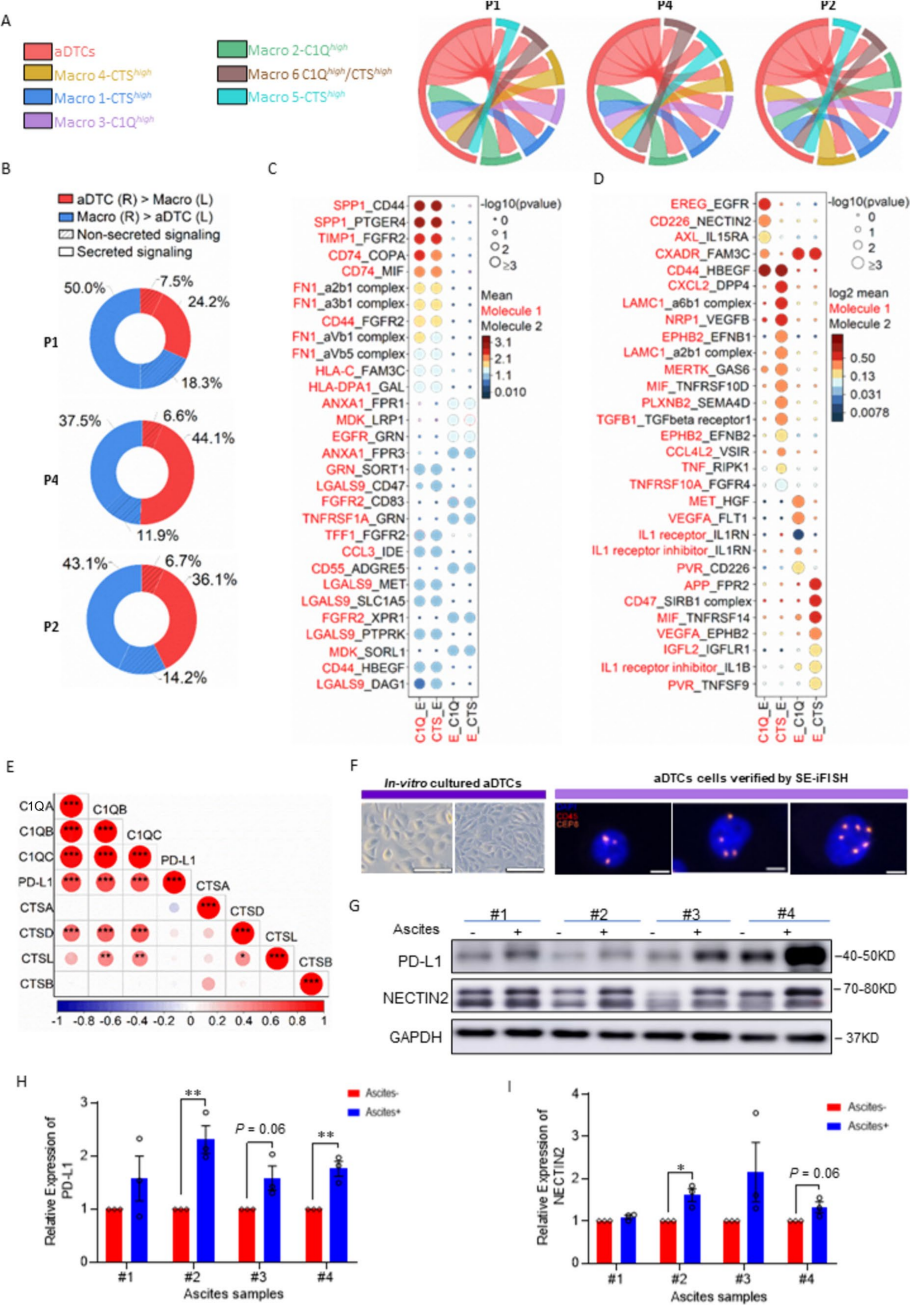
**C1Q**^***high***^** TAMs mediate the upregulation of PD-L1 and NECTIN2 on aDTCs**. **a** Chord diagrams representing cell–cell communication (CCC) between aDTCs and TAM clusters identified in Fig. 3e in individual ^*AS*^Subtype 1 ascites. Different colors on the outer circle of the chord diagram are correlated to different cell clusters. Chords curved inside the circle represent interactions between two cell clusters they connected. Wider chords mean stronger interactions. **b** Donut plots displaying proportions of secreted signaling and non-secreted signaling from aDTCs to TAMs (aDTCs (R) > TAMs (L)) or vice versa (TAMs (R) > aDTCs (L)) in each ^*AS*^Subtype 1 ascites sample. **c** Top 30 interaction pairs between C1Q^*high*^ (labeled as C1Q in the graph) or CTS^*high*^ (CTS) TAMs and aDTCs (Labeled as E). **d** Specific interaction pairs between C1Q^*high*^ (C1Q) or CTS^*high*^ (CTS) TAMs and aDTCs (Labeled as C1Q in the graph) or CTS^*high*^ (CTS) TAMs and aDTCs (E). In C and D, the sending cells and signals are labeled red. **e** Expressional correlations of C1Q or CTS genes to PD-L1 in 48 metastatic GC from TCGA database. Red means a positive correlation, and blue represents a negative correlation. Data are correlated using Pearson’s correlation coefficient. (^*^P < 0.05; ^**^P < 0.01; ^***^P < 0.001). **f** Representative imaging of in vitro cultured aDTCs. Left panels: The morphology of in vitro cultured aDTCs. Right panels: Identification of in vitro cultured aDTCs using SE-iFISH. The bars in the left panels are 200 µm, and those in the right panels are 5 µm. **g** Protein levels of PD-L1 and NECTIN2 on 4 in vitro cultured aDTCs with or without treatments of corresponding ascites supernatants (Ascites #1, #2, #3, and #4) detected by western blot. **h** and **i** Quantification of the band intensities of PD-L1 and NECTIN2 from (**g**). The band intensities are determined by the software ImageJ with GAPDH as the standard (Mean ± SD, *N* = 3, paired t—test was used for statistical analysis, ^*^P < 0.05; ^**^P < 0.01; ^***^P < 0.001; *N* = 3). (Abbreviations: *aDTC* ascites-disseminated tumor cells, *CTS* cathepsin, *MPs* mononuclear phagocytes, *TAM* tumor-associated macrophages, *UMAP* Uniform Manifold Approximation and Projection)

Having uncovered the specific CTS-to-C1Q transition in ascitic TAMs, we next investigated how the TAMs in different transitional states interact with aDTCs and influence their dissemination. To this end, the cell-to-cell communications (CCC) between TAMs and aDTCs were analyzed using the Cell-PhoneDB in an aDTC-rich ^*AS*^ Subtype 1 ascites. The interaction strength between TAM lineages and aDTCs is mapped in Fig. 4a and Supplementary Fig. S4E, which show that the interaction strength of aDTCs with CTShigh (Macro 1) and C1Qhigh (Macro 2) lineages is comparable (Fig. 4a). Similarly, a comparison of the interaction types and directionality between the CTS^*high*^ or C1Q^*high*^ lineages and aDTCs did not show any difference (Fig. 4b and Supplementary Fig. S4F). These results inferred that CTS^*high*^ and C1Q^*high*^ TAMs crosstalk robustly with aDTCs. CTS^*high*^ or C1Q^*high*^ TAMs are more likely the signal-sending cells propagating secreted signaling to modulate aDTC behavior.

Accordingly, we examined the secreted crosstalk between CTS^*high*^ or C1Q^*high*^ TAMs and aDTCs. Figure 4c, d displays the top 30 difference or specifically interaction pairs between TAM lineages and aDTCs. CTS^*high*^ and C1Q^*high*^ TAMs generate immunosuppressive interactions with aDTCs via predominantly secretion of SPP1 [35], FN1 [36], TIMP1 [37], or upregulation of CD74 [38], all of which foster tumor-cell metastasis (Fig. 4c). Specifically, CTS^*high*^ TAMs are inclined to secrete CXCL2 [39], LAMC1 [40], and NRP1 [41], which shape the TME of pre-metastatic niches and facilitate angiogenesis [42, 43]. Additionally, CTS^*high*^ TAMs secrete EPHB2, which contributes crucially to tumor-cell stemness via engagement to its receptor EFNB1 [44]. C1Q^*high*^ TAMs interact principally with aDTCs via the epiregulin-epidermal growth factor receptor (EGFR) [45] and CD226-NECTIN2 receptor–ligand pairs, which are involved in tumor-cell survival and proliferation [46, 47] (Fig. 4d).

Collectively, we suggested that CTS^*high*^ TAMs likely regulate the initial phases of aDTC dissemination via programming pre-metastatic TME and attracting aDTCs homing into ascites, while the stepwise transition from CTS^*high*^ to C1Q^*high*^ TAMs triggers and maintains the overproliferation of homed aDTCs in ascites.

### C1Q^***high***^ TAMs drive aDTC immune evasion in the proliferative phase of ascitic metastasis

**Fig. 5:**
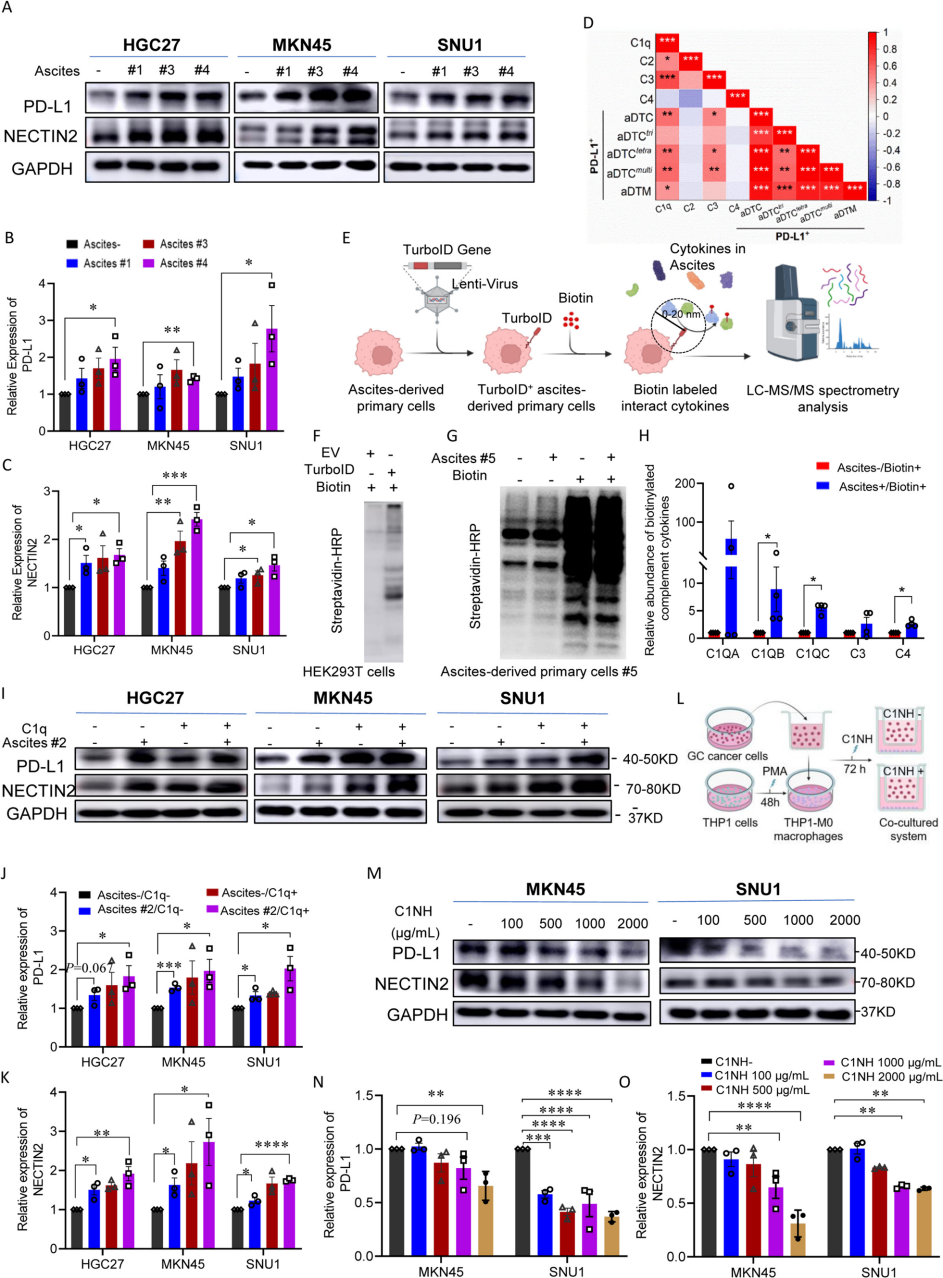
**C1q participates in PD-L1 and NECTIN2 upregulation on aDTCs**. **a** Protein levels of PD-L1 and NECTIN2 on 3 GC cell lines (HGC27, MKN45, and SNU1) with or without treatments of ascites supernatants (Ascites #1, #3, and #4) detected by western blot. **b** and **c** Quantification of the band intensities of PD-L1 (**b**) and NECTIN2 (**c**) from (**a**). **d** Correlations between protein levels of complement proteins C1q, C2, C3, and C4 detected by ELISA and the enumeration of PD-L1^+^ aDTCs or aDTMs in the SE-iFISH cohort. Red means a positive correlation, and blue represents a negative correlation. Data are correlated using Pearson’s correlation coefficient. (^*^P < 0.05; ^**^P < 0.01; ^***^P < 0.001). **e** Workflow diagrams of TurboID biotin labeling system for interaction proteomics. TurboID genes are transfected into in vitro cultured aDTCs with lentivirus. Overexpressed TurboID in the transfected aDTCs can biotinylate neighboring proteins in ascites supernatant interacting with aDTCs. Then, the biotinylated proteins are isolated by streptavidin-coated beads and analyzed using LC–MS/MS. **f** Western blots showing efficient biotinylation in HEK293T transfecting with TurboID vector compared with those with empty vector (EV). **g** Western blots showing biotinylated proteins enriched from in vitro cultured aDTCs treated with or without paired ascites supernatant in the presence or absence of biotin. **h** Histogram showing the relative abundance of C1QA, C1QB, C1QC, C3, and C4 in biotinylated proteins enriched from in vitro cultured aDTCs treated with or without pairing ascites. Levels of complement components in enriched proteins are detected using LC–MS/MS, and fold changes are used to display the relative abundance of complement proteins. (Mean ± SD, *N* = 4, Mann–Whitney test was used for statistical analysis. *P < 0.05). **i** Protein levels of PD-L1 and NECTIN2 on 3 GC cell lines (HGC27, MKN45, and SNU1) treated with or without ascites #2 after adding recombinant complement C1q cytokines. **j** and **k** Quantification of the band intensities of PD-L1 (**j**) and NECTIN2 (**k**) in (**i**). **l** Schematic diagram showing co-culturing of gastric cancer cell lines with THP-1 derived macrophages in the presence or absence of human recombinant C1 inhibitor (C1NH). **m** Expression of PD-L1 and NECTIN2 on MKN45 and SNU1 cell lines co-cultured with THP-1 derived macrophages with different concentrations of C1NH. **n** and **o** Quantification of the band intensities of PD-L1 (**n**) and NECTIN2 (**o**) in (**m**). All the bands intensities are determined by the software ImageJ with GAPDH as the standard (Mean ± SD, *N* = 3, two-way ANOVA test was used for statistical analysis, ^*^P < 0.05; ^**^P < 0.01; ^***^P < 0.001). (Abbreviations: *aDTC* ascites-disseminated tumor cells, *LC–MS/MS* liquid chromatography-mass spectrometry/mass spectrometry)

Due to the crucial role of the CTS-to-C1Q transition in the TAM-aDTC crosstalk, we next investigated whether this transition was also involved in the aDTC PD-L1-mediated immune escape, as demonstrated in the SE-iFISH cohort (Fig. 1). By analyzing RNA-seq data of 48 metastatic GCs from The Cancer Genome Atlas (TCGA) database, we first noted that C1Q genes (C1QA, C1QB, and C1QC), rather than CTS genes (CTSA, CTSB, CTSD, and CTSL), showed a positive correlation with the PD-L1 levels (Fig. 4e). To confirm further the involvement of C1Q^*high*^ TAMs in the PD-L1 upregulation on aDTCs, we cultured aDTCs from eight ascites of PM-GC samples in vitro (Fig. 4f). Treatments of the ascitic supernatants significantly enhanced PD-L1 expression on the in vitro cultured aDTCs (Fig. 4g, h and Supplementary Fig. S4G-H). Similarly, NECTIN2, which interact specifically with C1Q^*high*^ TAMs, expressions were upregulated in ascites-treated aDTCs (Fig. 4g, i, Supplementary Fig. S4G and S4I). This synchronous upregulation of PD-L1 and C1Q^*high*^ TAM-interacting proteins was also observed in ascites-treated GC cell lines HGC27, MKN45, and SNU1 (Fig. 5a–c), confirming that PD-L1 upregulation on aDTCs occurs in the proliferative phase of ascitic metastasis, coincident with the C1Q^high^ TAMs-driven overproliferation of aDTCs.

### Complement protein C1q participates in PD-L1 upregulation on aDTCs driven by C1Q^***high***^ TAMs

Next, we investigated whether C1Q^*high*^ TAMs modulate PD-L1 and NECTIN2 overexpression on aDTCs by secreting complement protein C1q in the SE-iFISH cohort. The levels of C1q and C3 in ascites both showed a positive correlation with the number of PD-L1^+^ aDTCs and aDTMs (Fig. 5d), suggesting that the activation of the appropriate classical complement pathway underlies PD-L1 upregulation on aDTCs.

To further illustrate the direct interaction of C1q and ascitic tumor cells, the biotin ligase TurboID labeling system [28] was transfected into in vitro cultured aDTCs (Fig. 5e). The overexpressed TurboID biotinylates neighboring cytokines in ascites supernatant that interacts with aDTCs. Further proteomic analysis of the biotinylated cytokines enriched by streptavidin magnetic beads (Fig. 5f, g) showed that complement proteins C1QA, C1QB, C1QC, C3, and C4 were all enriched in ascites-treated cells (Fig. 5h), demonstrating the direct crosstalk between components in the complement pathway and ascitic tumor cells.

The next question is whether the engagement of complement proteins influences PD-L1 and NECTIN2 expression on ascitic tumor cells. To address this, we compared PD-L1 and NECTIN2 expressions on GC cell lines treated with either singular ascitic supernatants or their combination with the recombinant C1q protein. Adding recombinant complement C1q protein to GC cell lines HGC27, MKN45, and SNU1 treated by ascites #2 can further augment PD-L1 and NECTIN2 upregulation induced by singular ascitic supernatants (Fig. 5i–k).

To further confirm the role of C1Q^*high*^ TAMs, GC cell line MKN45 and SNU1 were co-cultured with THP-1 derived macrophages in the presence or absence of C1NH, the human recombinant C1 inhibitor used to abolish the function of secreted C1q from macrophages (Fig. 5l). As shown in Fig. 5m–o, addition of C1NH significantly mitigates both PD-L1 and NECTIN2 expression on MKN45 and SNU1, which further confirms that C1q secreted by co-cultured macrophages mediates the activation of complement pathway, participating in the PD-L1 and NECTIN2 upregulation and promoting aDTCs immune evasion in PM-GC.

### CTS-to-C1Q transition of TAMs fosters therapeutic resistance of PM-GC

**Fig. 6:**
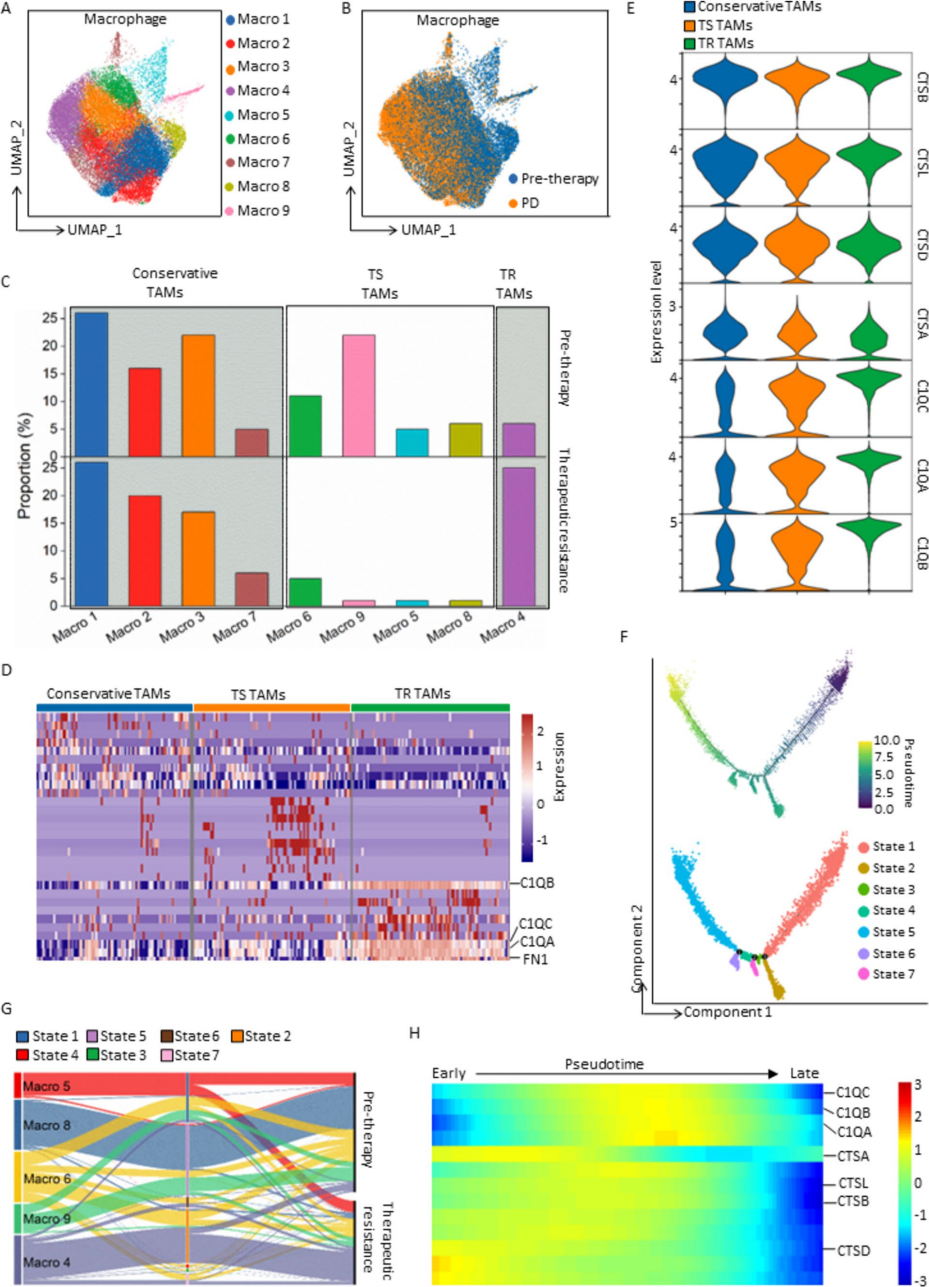
**scRNA-seq analysis of the TAMs from the treatment-naïve and paired therapeutically resistant ascites of eight patients with PM-GC in the scRNA-seq validation cohort**. **a** and **b** UMAP representation of unsupervised clustered macrophage lineages from all treatment-naïve and therapeutically resistant ascites. Different colors in (**a**) are correlated to different macrophage lineages, and those in (**b**) represent pre-therapeutic or resistant (progressive disease, PD) ascites. **c** Proportional variations of each TAM cluster after therapeutic resistance, categorizing TAM clusters into three subtypes: conservative TAMs (the proportions are almost unvaried after therapeutic resistance), therapeutically sensitive (TS) TAMs (clusters that can be reduced after therapy), and therapeutically resistant (TR) TAMs (the proportion elevated after therapeutic resistance). **d** Top 10 DEGs identified in conservative, TS, and TR TAMs. All genes shown in this heatmap are listed in detail in Supplementary Table 6. Each column represents a unique cell, and each row represents the normalized expression of each gene. **e** Expression levels of CTS and C1Q genes in conservative, TS, and TR TAMs. **f** Trajectory analysis of TAM differentiation following therapeutic resistance. Trajectories are, respectively, colored according to pseudotime (upper panel) and developmental states (lower panel). **g** Sankey diagram displaying the dynamic evolution of dominant TS (Macro 5, 8, 6, and 9) and TR TAMs (Macro 4) following therapeutic resistance. Nodes with different colors in the left and middle columns represent different TAM lineages and developmental states, respectively. **h** Variations of CTS and C1Q genes following pseudotime. (Abbreviations: *CTS* cathepsin, *DEGs* differentially expressed genes, *TAM* tumor-associated macrophages, *TME* tumor microenvironment, *UMAP* Uniform Manifold Approximation and Projection)

Having determined the contribution of the CTS-to-C1Q transition of TAMs to aDTC dissemination and immune escape, we next investigated whether this transition could lead to therapeutic resistance in patients with PM-GC. To validate the CTS-to-C1Q transition of ascitic TAMs specifically, we used another independent cohort containing both treatment-naïve and paired therapeutically resistant ascites samples from eight patients with PM-GC (scRNA-seq validation cohort, Supplementary Fig. S1 and Supplementary Table S5). Figure 6a displays TAM lineages clustered from 18 ascites samples from the scRNA-seq validation cohort. Comparison of TAM lineages from treatment-naïve or therapeutically resistant ascites patients highlights cluster Macro 4 as the only therapeutically resistant (TR) TAM lineage, which was determined by its proportional elevation in the therapeutically resistant ascites (Fig. 6b, c and Supplementary Fig. S5A). Transcriptional levels of C1QA, C1QB, and C1QC showed a marked increase in Macro 4 (Fig. 6d, e). Conversely, neither of the CTS genes showed variations in Macro 4 compared with the other TAM lineages (Fig. 6e).

Next, we investigated whether C1Q^*high*^ TR TAMs transitioned from the CTS^*high*^ lineages, leading to therapeutic resistance. Figure 6f shows the trajectory of the TAM differentiation following therapeutic resistance. The therapeutically sensitive (**TS**) Macro 5, identified by its large decrease in TR ascites level (Fig. 6c), is predominant in the TAM precursor lineage, presenting in the initial state (State 1 in Fig. 6f and Supplementary Fig. S5B-C). Without treatments, Macro 5 directly develops to another TS lineage, Macro 8, existing mainly in State 5 (Fig. 6g and Supplementary Fig. S5B-D). Other TS TAM lineages (Macro 6 and Macro 9) showed progression similar to that in State 5 (Fig. 6g). However, under therapeutic pressures, Macro 5 differentiates distinctly into the TR TAM lineage Macro 4 at the bifurcated States 2, 6, and 7 (Fig. 6f, g and Supplementary Fig. S5D). Further insight into the variations in C1Q and CTS gene levels following pseudotime shows that the CTS genes showed high expression in the initial pseudotime, when TS lineages were predominant. However, this overexpression of the CTS genes gradually transitioned to an overexpression of the C1Q genes at the intermediate pseudotime when the TR TAM lineage was developed (Fig. 6h).

Collectively, the results demonstrated that the TAM transition from the CTS^*high*^ to C1Q^*high*^ lineages facilitates the development of therapeutic resistance and ascitic progression in patients with PM-GC.

### Ascitic CD8^+^ T cells are dysfunctional, showing limited cytotoxic activity

**Fig. 7:**
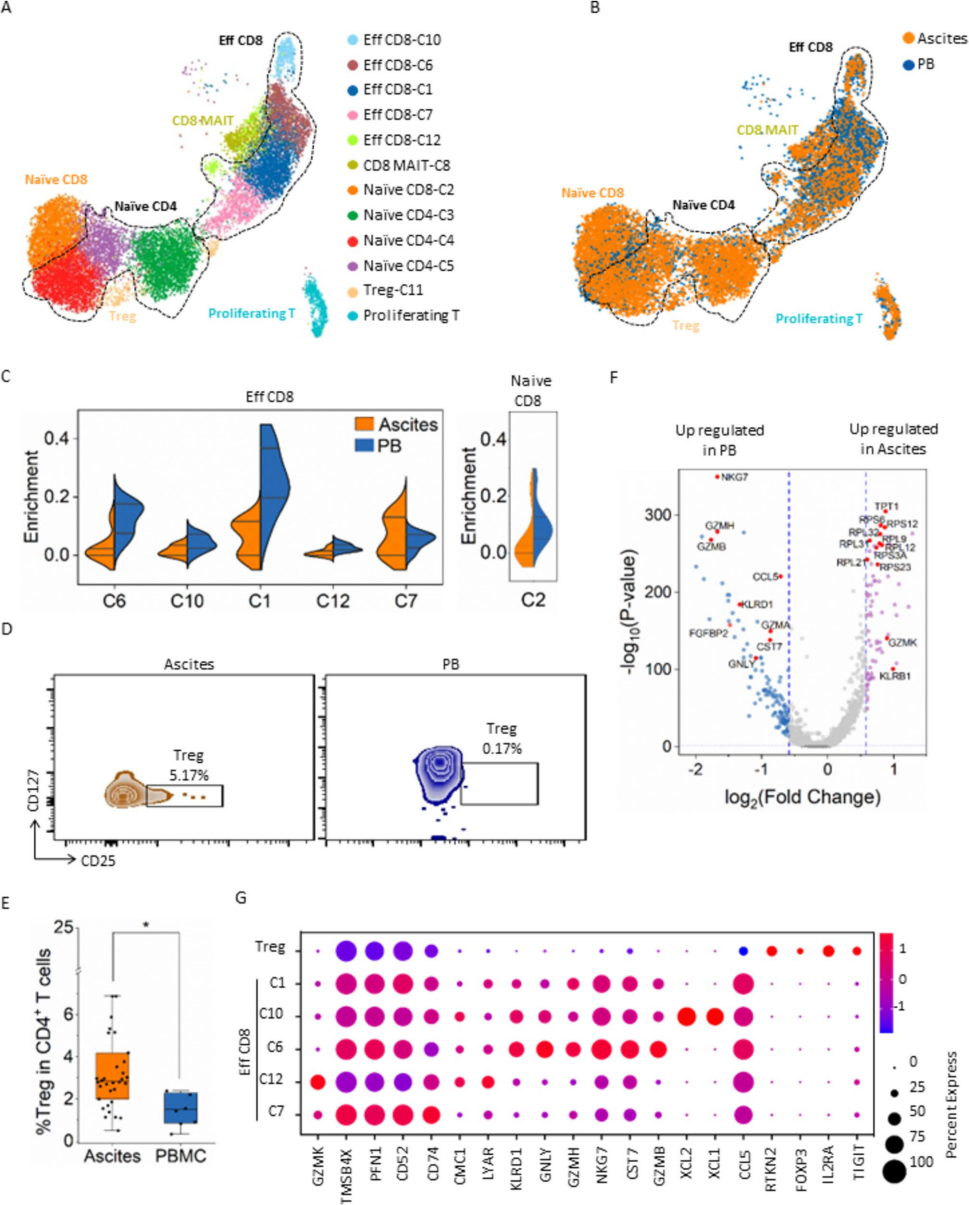
**scRNA-seq analysis of the T cells in seven treatment-naïve ascites and the paired peripheral blood samples from the scRNA-seq test cohort**. **a** and **b** UMAP representation of unsupervised clusters of T cells and their annotation in ascites and paired peripheral blood (PB) samples. Graphs are colored according to cell cluster (**a**) or sample type (**b**). **c** Comparison of the enrichment of each CD8^+^ T-cell cluster in ascites and paired PB samples. **d** Representative FACS plots of CD25^+^/CD127^−/low^ Treg cells in ascites and paired PB samples. Treg cells are gated by CD3^+^/CD4^+^/CD25^+^/CD127^−/low^. **e** Box chart displaying the proportion of Treg cells in CD4^+^ T cells in ascites or PB mononuclear cells (PBMC). **f** Volcano plot showing significantly upregulated genes in effector CD8^+^ T cells from ascites and PB samples. **g** Selected DEGs in the Treg cluster and each effector CD8^+^ T-cell cluster

As mentioned previously, the exclusive infiltration of T cells in ^*AS*^Subtype 3 ascites suggests their independent function in ascites development. Therefore, we explored the specific characteristics of ascitic T cells based on our scRNA-seq data from the scRNA-seq test cohort (seven treatment-naïve ascites and paired PB samples, Supplementary Fig. S1 and Supplementary Table S3). Overall, 12 T-cell clusters were identified in the ascites and paired PB samples (Fig. 7a and Supplementary Fig. S6A). The most variant lineages between ascites and PB were the effector CD8 T-cell clusters (Fig. 7b). The effector CD8 T-cell subpopulations Eff CD8 C6, C10, and C1 in ascites reduced quantitatively compared with those in PB. Conversely, the Eff CD8-C7 proportion showed significant elevation, especially in ^*AS*^Subtype 3 ascites, compared with that in PB (Fig. 7c and Supplementary Fig. S6B).

Besides the effector CD8 T cells, CD25^+^/CD127^low/−^ Treg cells were observed to infiltrate differentially in ascites and PB samples in the fluorescence-activated cell sorting analysis of ascites in the SE-iFISH cohort. The CD25 + /CD127low/- Tregs proportion was significantly higher in ascites than in PB (Fig. 7d, e).

Due to the heterogeneity of effector CD8 T-cell subsets, we next compared DEGs of effector CD8 T cells from ascites and PB samples. The genes relevant to T-cell-mediated cytotoxicity, including NKG7, GZMH, GZMB, and GNLY, were downregulated in ascitic CD8^+^ T cells. Inversely, TPT1, GZMK, KLRB1, and ribosomal protein-associated genes were upregulated (Fig. 7f). Further examination of DEGs from the individual effector CD8 T-cell clusters confirmed that Eff CD8-C7, which were enriched mainly in ascites, lacked cytotoxicity-associated gene expression. However, they presented higher GZMK expression than did Eff CD8-C1, C10, and C6, which existed predominantly in PB (Fig. 7g and Supplementary Fig. S6C). GZMK has a high expression on precursor-exhausted T cells [48]. Moreover, KLRB1 and TPT1 upregulation indicated a reduction in the cytotoxicity of CD8 T cells [49, 50]. Therefore, we conclude that the CD8^+^ T cells infiltrating in ascites are “senseless CD8^+^ T cells,” which have lost their antitumor capability.

## Discussion

Here, the heterogenous immune TME of ascites from patients with PM-GC was investigated according to three ascites subtypes: ^*AS*^Subtype 1, characterized as aDTC-rich ascites with an MP-dominant TME; ^*AS*^Subtype 2, with the same MP-dominant TME, but with a lower aDTC level than that of ^*AS*^Subtype 1; and ^*AS*^Subtype 3, with exclusive T-cell infiltration in the TME. For the first time, in TAM-dominant ascites, we identified a specific CTS-to-C1Q transition in ascitic TAMs, prompting aDTC dissemination and immune escape. CTS^*high*^ TAMs were identified as precursors for the initial construction of the pro-metastatic TME and to facilitate aDTCs homing in ascites. The later transitioned C1Q^*high*^ TAMs were of a terminal lineage that promoted the overproliferation, PD-L1-mediated immune evasion, and therapeutic resistance of the homed aDTCs in ascites. Moreover, in T-cell-dominant ascites, increased T-cell infiltration did not infer an “immune-hot” TME. The effector CD8^+^ T cells enriched with ascites were predominantly precursor-exhausted CD8^+^ T cells with limited antitumor cytotoxicity.

C1Q^*high*^ TAMs are immunosuppressive components in clear-cell renal cell carcinoma, hepatocellular carcinoma, breast cancer, and osteosarcoma [51–54]. However, the development of C1Q^*high*^ TAMs and mechanisms underlying their immunosuppression remain unclear. Here, we demonstrated that C1Q^*high*^ TAMs in ascites from patients with PM-GC are not pre-existing but are terminally differentiated from CTS^*high*^ TAM precursors. Regarding the immunosuppressive mechanisms, we highlighted the interaction between C1Q^*high*^ TAMs and aDTCs, showing that C1Q^*high*^ TAMs can trigger the PD-L1-mediated immune escape of aDTCs via secreted signaling, including C1q, and the associated complement pathway activation. Concurrently, C1Q^high^ TAMs improve the overproliferation of aDTCs via the indirect upregulation of NECTIN2 and EGFR. Besides driving the pro-metastatic process, C1Q^*high*^ TAMs induce therapeutic resistance in patients with PM-GC. TR TAM lineages all harbor significantly high expression of the C1Q genes.

The characteristics and functions of T cells infiltrating the ascites of PM-GC remain unknown. Our study is the first to suggest that the effector CD8^+^ T cells, predominantly enriched in T-cell-dominant ascites, harbored the upregulated GZMK, which was recently identified as a marker of the precursor-exhausted T cells in non-small-cell lung cancer [45]. Ascitic GZMK^+^CD8^+^ T cells in PM-GC lack the expression of cytotoxicity-associated genes, indicative of their loss of antitumor function. However, due to the small sample size, this study did not explore the mechanisms underlying the development of T-cell-dominant ascites were not explored in this study due to the small sample size. Limited aDTCs were detected in T cell-dominant ascites compared with that in MP-dominant ascites, suggesting that ascitic T cells may not interact primarily with the aDTCs. Further investigation on how the GZMK^+^CD8^+^ T cells in ascites contribute to ascites development still needs to be conducted.

In conclusion, our data have deepened the current understanding of the immune TME of ascites in PM-GC. We highlighted the interplay between TAMs and aDTCs and uncovered the mechanisms underlying the ascitic metastasis driven by the CTS-to-C1Q TAM transition.

### Supplementary Information

Below is the link to the electronic supplementary material.

Supplementary file1 (DOCX 2936 KB)

## Data Availability

All other data are available in the article and its Supplementary files or from the corresponding author upon reasonable request. Source data are provided with this paper. Further information and reasonable requests for resources and reagents should be directed to and will be fulfilled by the lead contact, Lin Shen (shenlin@bjmu.edu.cn).

## Abbreviations

aDTMs: Ascites-disseminated microemboli
aDTCs: Ascites-disseminated tumor cells
CCC: Cell-to-cell communications
C1NH: Human recombinant C1 inhibitor
C1Q: Complement 1q
CNV: Copy number variations
CTMs: Circulating tumor microemboli
CTCs: Circulating tumor cells
CTS: Cathepsin
CTSL: Cathepsin L
CSC: Cancer stem cell
DEG: Differentially expressed gene
EGFR: Epiregulin-epidermal growth factor receptor
ELISA: Enzyme-linked immunosorbent assay
FVF: Find variable features
GC: Gastric cancer
HIPEC: Hyperthermic intraperitoneal chemotherapy
LC–MS/MS: Liquid chromatography-mass spectrometry/mass spectrometry
MSI-H: Microsatellite instability-high
OS: Overall survival
PB: Peripheral blood
PBMCs: Peripheral blood mononuclear cells
PBS: Phosphate-buffered saline
PM: Peritoneal metastasis
PMA: Phorbol-12-myristate-13-acetate
PM-GC: GC with peritoneal carcinomatosis
PVDF: Polyvinylidene fluoride
RBCs: Red blood cells
RCLB: RBC lysis buffer
RNA-seq: Bulk transcriptional sequencing
SDs: Standard deviations
scRNA-seq: Single-cell RNA sequencing
SE-iFISH: Subtraction enrichment and immunostaining-fluorescence in situ hybridization
TAMs: Tumor-associated macrophages
TGF-β: Transforming growth factor-β
TGF-β1: Transforming growth factor-β1
TME: Tumor microenvironment
TCGA: The Cancer Genome Atlas
TR: Therapeutically resistant
TS: Therapeutically sensitive
UMAP: Uniform Manifold Approximation and Projection
UMI: Unique molecular identifier
