# Supplementary material for: Specific lineage transition of tumor-associated macrophages elicits immune evasion of ascitic tumor cells in gastric cancer with peritoneal metastasis
Source: Gastric Cancer. 2024 Mar 9;27(3):519–38. doi: 10.1007/s10120-024-01486-6 (PMC11016508; doi:10.1007/s10120-024-01486-6)
Supplement: Supplementary file 1 — Supplementary file1 (DOCX 2936 KB) [file 10120_2024_1486_MOESM1_ESM.docx]

**SUPPLEMENTARY MATERIALS AND METHODS**

**SUPPLEMENTARY FIGURES**

**
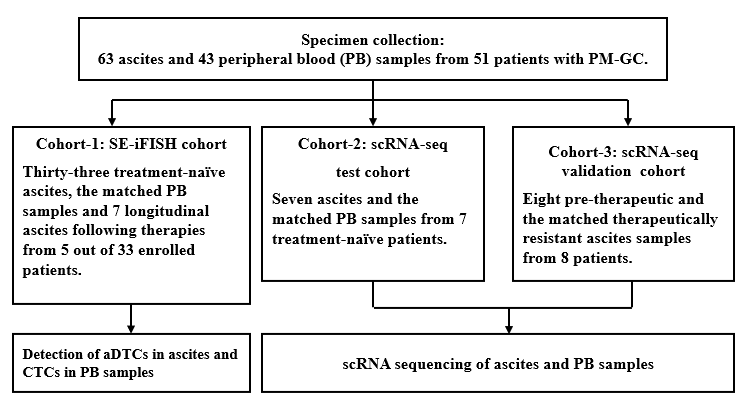
**

**Supplementary Figure S1. Schematic of the specimen collection of three independent cohorts used in this study**


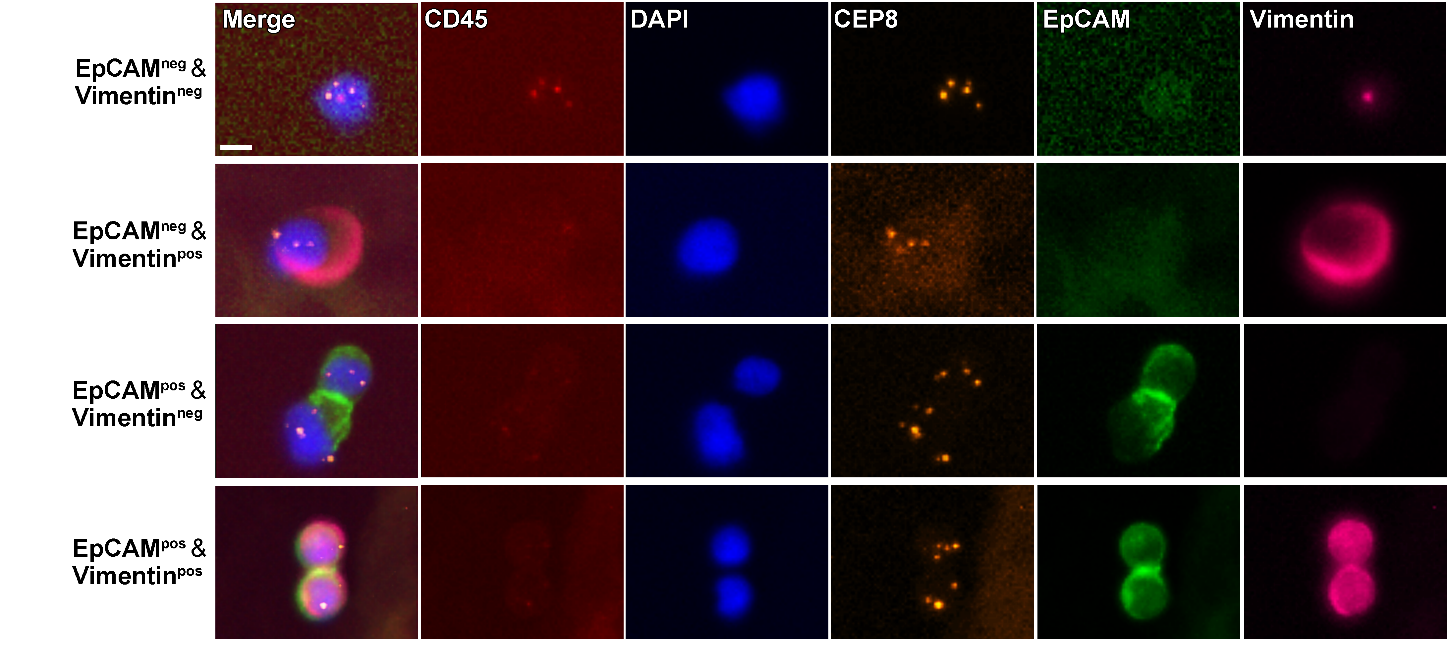


**Supplementary Figure S2. Fluorescent images showing heterogeneous expressions of EpCAM (Epithelial cell marker) and Vimentin (Mesenchymal cell marker) on aDTCs**. The bar is 5 µm.

**
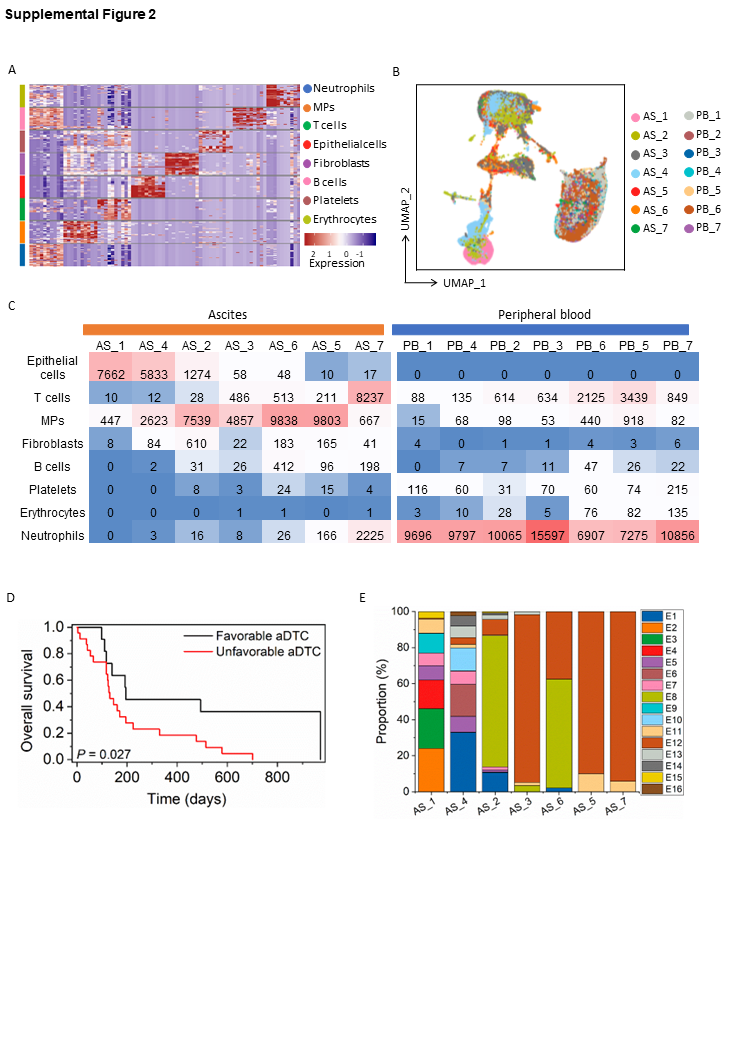
**

**Supplementary Figure S3. Comparison of the annotated cell clusters from the scRNA-seq of seven treatment-naïve ascites and the paired peripheral bloods in the scRNA-seq test cohort. (A)** The top 10 differentially expressed genes (DEGs) within the identified cell clusters. For a detailed list of these genes, refer to Supplemental Table 4. **(B)** UMAP of the annotated cell clusters obtained from seven treatment-naïve ascites and their paired peripheral blood, color-coded by individual samples. **(C)** Cell numbers within each annotated cell cluster for each sample processed via scRNA-seq. **(D)** Kaplan–Meier plots displaying the overall survival rates for patients characterized by either favorable aDTC counts (aDTCs < 5 cells/10 mL) or unfavorable aDTC counts (aDTCs ≥ 5 cells/10 mL). **(E)** Distribution of each epithelial cell cluster, as determined by unsupervised learning, in each ascites sample.


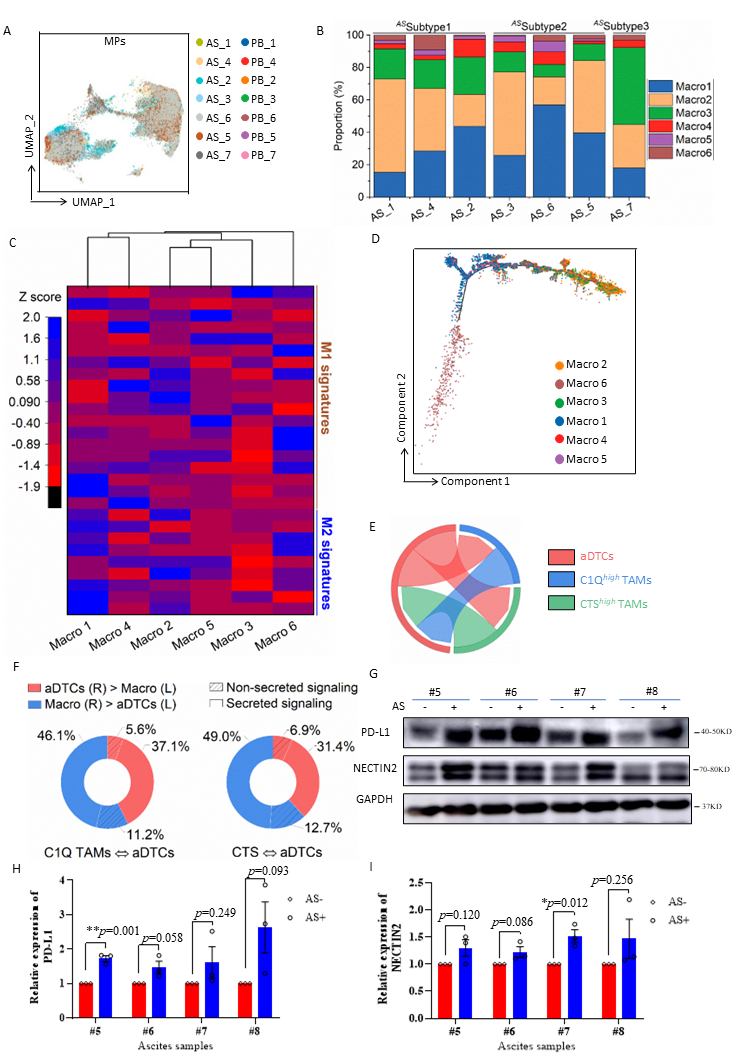


**Supplementary Figure S4. Identification of the CTS- to-C1Q transition of the ascitic TAMs and its involvement in the development of ascites. (A)**The UMAP of the MP subpopulation colored according to different samples. **(B)** Distribution of each TAM cluster from unsupervised learning in individual ascites samples. **(C)** Average expression of M1 and M2 signature genes in individual TAM clusters. Each column represents a unique gene, and each row represents a specific ascites sample. For a detailed gene list, see Supplemental Table 6. **(D)** The trajectory of TAM differentiation colored according to TAM clusters. **(E)** Chord diagram showing the interactions between aDTCs and C1Q*^high^* or CTS*^high^* TAMs in *^AS^*Subtype1 ascites. **(F)** Donut plots showing proportions of secreted signaling and non-secreted signaling between aDTCs and either C1Q*^high^* or CTS*^high^* TAMs in *^AS^*Subtype 1 ascites. **(G)** Protein levels of PD-L1 and NECTIN2 on four *in vitro* cultured aDTCs with or without exposure to corresponding ascites supernatants (ascites #5, #6, #7, and #8), as detected by Western blot. **(H and I)** Quantitation of the band intensities of PD-L1 and NECTIN2 from **(G)**. Band intensities were measured using the ImageJ software, with GAPDH serving as a standard (*P < 0.05; **P < 0.01; ***P < 0.001; *N* = 3).


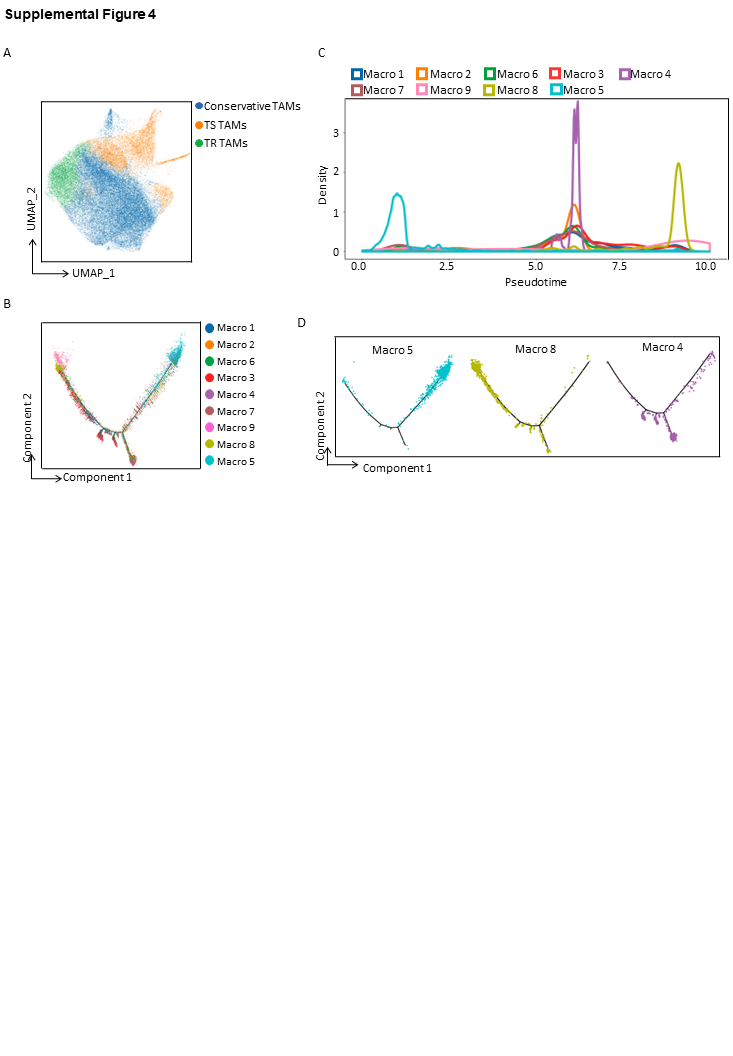


**Supplementary Figure S5. Differentiation of TAMs following therapeutic resistance.** **(A)** The UMAP representation of TAM clusters derived from both treatment-naïve and paired therapeutically resistant ascites from the scRNA-seq validation cohort. Cell clusters are color-coded based on their differential responses to treatments, defining three distinct TAM lineages: conservative (clusters remaining unchanged post-therapeutic resistance), therapeutically sensitive (TS, clusters significantly reduced post-therapies), and therapeutically resistant (TR, clusters increased post-treatment resistance). **(B)** The trajectory of TAM differentiation following therapeutic resistance, colored according to different TAM clusters determined through unsupervised learning. **(C)** Changes in the density of each TAM cluster over pseudotime. **(D)** The projection of TAM cluster Macro 5 (predominantly conservative TAMs), Macro 8 (predominantly TS TAMs), and Macro 4 (TR TAMs) on the trajectory of TAM differentiation following treatments.


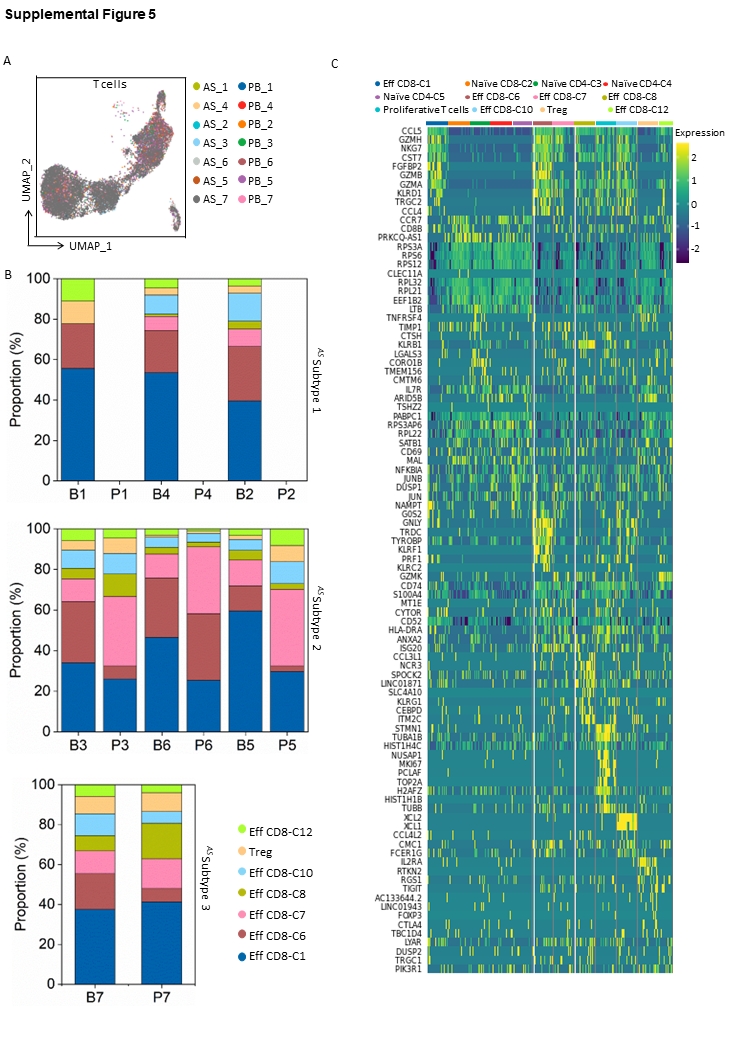


**Supplementary Figure S6. Stratification of T cells in treatment-naïve ascites.** **(A)** The UMAP representation of unsupervised clustering of T cells, derived from seven treatment-naïve ascites and their corresponding peripheral blood (PB), as part of the scRNA-seq test cohort. Cell clusters are color-coded based on individual samples. **(B)** The distribution of subsets of effector CD8+ T cells and regulatory Tregs within each subtype of ascites. **(C)** Heatmap presenting the top 10 DEGs within each distinct T cell cluster.

**SUPPLEMENTARY TABLES**

| **Supplementary Table S1**  **Markers used to annotate cell clusters in Figure 2A** | | | |
| --- | --- | --- | --- |
| **Cell type** | **Markers** |  |  |
| T cells | CD2, CD3D, TRAC, TRBC2 |  |  |
| B cells | CD79A, CD79B, MS4A1, JCHAIN, MZB1 |  |  |
| MPs | LYZ, CD14, VCAN, C1QA, CD68, CDC |  |  |
| Erythrocytes | HBB, HBA1, ALAS2, SNCA, CA1 |  |  |
| Neutrophils | CSF3R, CXCR2, FCGR3B, LCN2, CAMP |  |  |
| Fibroblasts | DCN, COL1A1, COL1A2 |  |  |
| Epithelial cells | EPCAM, CLDN4, CLDN7, TFF3, REG4 |  |  |
| Platelets | PPBP, TUBB1, PF4 |  |  |

| **Supplementary Table S2**  **Clinical characteristics of patients with PM-GC in the SE-iFISH cohort (Cohort-1, *N* = 33)** | |
| --- | --- |
| **Characteristics*** | **No. (%)** |
| Sex |  |
| Male | 18 (54.5%) |
| Female | 15 (45.5%) |
| Age (years) |  |
| <60 | 21 (63.6%) |
| ≥60 | 12 (36.4%) |
| Location of the primary tumor |  |
| Non-GEJ | 28 (84.8%) |
| GEJ | 5 (15.2%) |
| Lauren classification |  |
| Intestinal | 8 (24.2%) |
| Diffuse | 19 (57.6%) |
| Mixed | 5 (15.2%) |
| Unknown | 1 (3.0%) |
| PD-L1 score |  |
| CPS <5 | 29 (87.9%) |
| CPS ≥5 | 4 (12.1%) |
| HER2 score |  |
| ≤1+ | 28 (84.8%) |
| 2+/FISH- | 3 (9.1%) |
| 2+/FISH+ | 0 (0.0%) |
| 3+ | 2 (6.1%) |
| MSI type |  |
| MSI-H | 3 (9.1%) |
| MSS | 29 (87.9%) |
| Unknown | 1 (3.0%) |
| **Abbreviations:* GEJ: Esophagogastric junction; CPS: Combined positive score; MSI: Microsatellite instability; MSS: Microsatellite stability; MSI-H: Microsatellite instability-high. | |

| **Supplementary Table S3**  **Clinical characteristics of patients with PM-GC in the scRNA-seq test cohort**  **(Cohort-2, *N* = 7)** | | | | | | | |
| --- | --- | --- | --- | --- | --- | --- | --- |
| **Sample ID** | **AS_1** | **AS_2** | **AS_3** | **AS_4** | **AS_5** | **AS_6** | **AS_7** |
| Location of the primary tumor | Non-GEJ | Non-GEJ | Non-GEJ | Non-GEJ | Non-GEJ | Non-GEJ | GEJ |
| Lauren classification | Diffuse | Diffuse | Intestinal | Mixed | Diffuse | Diffuse | Intestinal |
| MSI type | MSS | MSS | MSS | MSS | MSS | MSS | MSS |
| CPS Score  of PD-L1 | 1 | 5 | 10 | 0 | <1 | 1 | 0 |
| HER2 Score | 0 | 1+ | 0 | 0 | 0 | 3+ | 1+ |

**Abbreviations:* GEJ: Esophagogastric junction; CPS: Combined positive score; MSI: Microsatellite instability; MSS: Microsatellite stability; MSI-H: Microsatellite instability-high.

| **Supplementary Table S4**  **Signature genes of M1 and M2 macrophages in extend data Figure 2C** | |
| --- | --- |
| **M1 signatures** | **M2 signatures** |
| SLC2A6 | CD302 |
| SLC31A2 | CD209 |
| IDO1 | MS4A6A |
| PSME2 | MS4A4A |
| PSMB9 | CCL23 |
| APOL3 | CCL18 |
| APOL1 | CCL13 |
| APOL2 | LIPA |
| APOL6 | MAF |
| IRF1 | — |

| **Supplementary Table S5**  **Clinical characteristics of patients with PM-GC in scRNA-seq validation cohort**  **(Cohort-3, *N* = 8)** | | | | | | |  |
| --- | --- | --- | --- | --- | --- | --- | --- |
| **Patient ID** | **Location of primary tumors** | **Lauren Classification** | **MSS type** | **CPS Score of PD-L1** | **HER2 Score** | **Treatments** |  |
|  |  |  |  |  |  |  |  |
| Patient 1 | Non-GEJ | Intestinal | MSS | 1 | 0 | S-1+ TNF + DDP |  |
| Patient 2 | Non-GEJ | Mixed | MSS | 30 | 1+ | Toripalimab + SOX |  |
| Patient 3 | Non-GEJ | Intestinal | MSS | <1 | 1+ | PTX + TNF |  |
| Patient 4 | Non-GEJ | Intestinal | Unknown | 50 | 0 | PEM + PTX + S-1 |  |
| Patient 5 | Non-GEJ | Diffuse | MSS | 40 | 1+ | Toripalimab + SOX |  |
| Patient 6 | Non-GEJ | Diffuse | MSS | 1 | 0 | Camrelizumab + Apatinib + PTX + S-1 |  |
| Patient 7 | Non-GEJ | Diffuse | MSS | <1 | 1+ | Toripalimab + Surufatinib + BEV |  |
| Patient 8 | Non-GEJ | Mixed | MSS | 0 | 0 | Toripalimab + Surufatinib + DDP |  |
| **Abbreviations:* TNF: Tumor necrosis factor; DDP: Cisplatin; SOX: S-1 + Oxaliplatin; PTX: Paclitaxel; PEM: Pemetrexed; BEV: Bevacizumab. | | | | | | |  |

| **Supplementary Table S6**  **Top10 DEGs in conservative, TS, and TR TAM clusters from Figure 6D** | |
| --- | --- |
| **TAM lineage** | **DEGs** |
| Conservative | S100A12, PID1, APOBEC3A, RETN, VCAN, CCR2, THBS1, CCL2, FCN1, CLEC5A |
| TS | TM4SF4, SPRR3, FGFR2, PRSS3, ANXA10, SDC1, CD24, TMC5, TCN1, OLFM4 |
| TR | C1QB, LYPD2, TIMD4, HOXB6, HAMP, RBP4, GPX3, C1QC, C1QA, FN1, |

**Abbreviations:* TS: Therapeutically sensitive; TR: Therapeutically resistant.

**SUPPLEMENTARY MATERIALS AND METHODS**

**Specimen collection**

The three independent cohorts are the SE-iFISH, scRNA-seq test, and scRNA-seq validation cohorts (**Supplementary Fig. S1**). A total of 33 patients with PM-GC were enrolled in the SE-iFISH cohort (Cohort-1). In addition to 33 treatment-naïve ascites samples, seven longitudinal ascites following therapies were also collected from 5 out of 33 patients. To detect ascites-disseminated tumor cells (aDTCs) / circulating tumor cells (CTCs). The scRNA-seq test cohort (Cohort-2) comprised seven ascites samples and matched peripheral blood samples from seven treatment-naïve patients (**Supplementary Table. S3**). Finaly, the scRNA-seq validation cohort (Cohort-3) comprised 16 ascites samples and matched paired therapeutically resistant ascites samples from eight treatment-naïve patients (**Supplementary Table. S5**).

**Sample collection and preparation for scRNA-seq**

Fresh ascites samples were filtered and centrifuged at 350 g for 5 min. Peripheral blood mononuclear cells (PBMCs) were isolated using Ficoll-Paque Plus medium (GE Healthcare) and washed with Ca/Mg-free Phosphate-buffered saline (PBS). GEXSCOPE^®^ RBC lysis buﬀer (RCLB, Singleron) was used to remove red blood cells (RBCs). The Dead Cell Removal Kit (Miltenyi Biotec, cat. no. 130-090-101, Germany) was used per the manufacturer’s protocol. Finally, the samples were stained with trypan blue, and cell quantity and viability were assessed under the microscope using a hemocytometer.

**Quality control, dimension reduction, and clustering**

For the scRNA-seq test cohort (Cohort-2), the cells were filtered using UMI counts of >30,000, gene counts of <200 or >5,000, and mitochondrial content of more than 50%. After filtering, 145,407 cells were retained for downstream analyses, with an average of 802 genes and 2245 UMIs per cell. For the scRNA-seq validation cohort (Cohort-3), in each sample dataset, we filtered the expression matrix using the following exclusion criteria: cells with a gene count <200 or with a top 2% gene count, cells with a top 2% UMI count, cells with mitochondrial content >30%, and genes expressed in less than five cells. After filtering, 136,154 cells were retained for downstream analyses, with an average of 1,949 genes and 6,583 UMIs per cell. The raw count matrix was normalized by the total counts per cell and transformed logarithmically into a normalized data matrix. Thereafter, we used the different functions of Seurat v3.1.2 (https://satijalab.org/seurat/) for dimension reduction and clustering. The Normalize Data and Scale Data functions were used to normalize and scale the gene expression, and the top 2,000 variable genes were selected using the Find Variable Features (FVF) function in principal component analysis. Using the top 20 principal components, we separated the cells into multiple clusters with the Find Clusters function.

**Cell lines**

Gastric cancer (GC) cell lines SNU1, MKN45, and HGC27 were obtained from the American Type Culture Collection (Manassas, VA, USA) and maintained in our laboratory. These cell lines were cultured at 37℃ with 5% CO_2_ in RPMI-1640 (Invitrogen, Carlsbad, CA, USA) containing 10% FBS (Gibco, Scoresby, Australia), 100 U/mL Penicillin-Streptomycin (P/S) (Gibco, Waltham, MA, USA). THP-1 (Human leukemia monocytic cell line) cells were purchased from American Type Culture Collection and maintained in our laboratory. Cells were cultured at 37°C with 5% CO_2_ in RPMI-1640 (Invitrogen, Carlsbad, CA, USA) containing 10% FBS and 100 U/mL P/S and 0.1% mM 2-mercaptoethanol (Gibco， Cat:21985023, USA). Using 100 ng/mL phorbol-12-myristate-13-acetate (PMA, Sigma-Aldrich) treated for 48h, the THP-1 cells were differentiated into M0 macrophages. HEK293T (Human Embryonic Kidney 293T) cells were purchased from American Type Culture Collection and maintained in our laboratory. Cells were cultured at 37°C with 5% CO_2_ in DMEM (Invitrogen, Carlsbad, CA, USA) containing 10% FBS and 100 U/mL P/S.

**In vitro culturing of ascitic-derived primary tumor cells**

Ascitic fluid was obtained from eight patients with PM-GC from the Peking University Hospital, and histopathologic and cytopathologic examinations were performed in the hospital’s Department of Pathology to confirm the diagnosis and ascertain tumor grade. Patients with newly diagnosed diseases who had not received any hyperthermic intraperitoneal chemotherapy (HIPEC) were included in the study. Before use, ascites samples were cultured in Iscove’s Modified Dulbecco’s Medium (Gibco, Waltham, MA, USA), supplemented with 10% FBS (Gibco, Scoresby, Australia), 100 U/mL P/S, and 1% (Gibco), at 37℃ and 5% CO2. The cell culture dish was coated with 1-2% Reduced Growth Factor Basement Membrane Extract, Type 2, Select (3536-005-02; R&D SYSTEMS BIO-TECHNE, Minneapolis, MN, USA).

**Construction of lentiviral vector expressing TurboID and its transfection**

Lentivirus expressing TurboID was produced by co-transfecting lentiviral vector pLVX containing genes expressing TurboID, and the lentiviral packaging plasmids psPAX2 (Addgene plasmid 12260, Addgene, Cambridge, MA, USA) into HEK293T cells. After 72-hour of transfection, cell debris in the media was removed by centrifugation at 2000 rpm for 10 min, and the TurboID lentivirus was concentrated by Lenti-X concentrator (Clonetech, Mountain View, CA, USA). The TurboID gene was inserted into an empty pLVX-tre3g-mcs vector constructed by Gibson cloning (CL116, Biomed, Beijing, China) to express TurboID. The coding sequences of this pLVX-TurboID vector were further verified by Sanger sequencing (RuiBiotech, Beijing, China).

For transfection, ascitic tumor cells in vitro cultured in 10 cm dishes were transfected at 70% confluency with TurboID lentivirus in the presence of 5 μg/mL polybrene for 48 h. The biotin blocking solution (BioLock, Cat. 2-0205-050, IBA Lifesciences, Göttingen, Germany BioLock) was added for 20–24 h to block initial biotinylated proteins in ascitic tumor cells, which was then replaced by fresh medium or paired ascites supernatants in the presence or absence of 500 mM biotin (Cat.HY-B0511/CS-2719, MedChemExpress). After 4 h treatments with or without ascites, total proteins in supernatants were firstly ultrafiltered using Ultracel-100K regenerated cellulose membrane centrifugal filters (Cat. UFC910096, Millipore, Billerica, MA, USA) and then enriched by streptavidin-coated magnetic beads for further mass spectrometry.

**Mass spectrometry data analysis**

The enrichment biotinylated protein samples mentioned above were detected by LC-MS/MS analysis in the Analytical Instrumentation Center of Peking University. For LC-MS/MS analysis, an Easy nLC 1000 system (Thermo Scientific, Waltham, WA, USA) was used to deliver the HPLC gradient. The eluted peptides were sprayed into a Velos Pro Orbitrap Elite mass spectrometer (Thermo Scientific, USA) equipped with a nano-ESI source, and the mass spectrometer was operated in data-dependent mode. Data processing was carried out using Thermo Proteome Discoverer 2.4 using a SwissProt Human Database (version 2017-10-25). Carbamidomethyl (Cys) was chosen as static modification, and oxidation (Met) was chosen as variable modification. The set mass tolerance was 10 ppm for precursor ions and 0.6 Da for fragmentions. The limit for missed cleavages was set at 2. Validation of peptide spectral matches (PSM) was conducted using the Percolator algorithm, adhering to q-values at a 1% FDR. In the case of label-free quantitation, protein abundances were determined by totaling the abundances of corresponding peptides post-normalization within each sample.

**Flow cytometric analysis**

Frozen PBMC or ascitic sediment cells were thawed and washed twice with a staining buffer (PBS with 5% FBS). The samples were subsequently stained with the Fixable Viability Stain 780 (FVS780) (Cat. 565388; BD Biosciences, San Jose, CA, USA) to discriminate between live and dead cells. The samples were washed and incubated with human surface marker antibodies.

**Antibodies used in flow cytometric analysis**

| ANTIBODIES | SOURCE | IDENTIFIER |
| --- | --- | --- |
| BUV 395 Mouse Anti-Human CD3 | BD Horizon^TM^ | Cat # 563546/563548 |
| PE-CF594 Mouse Anti-Human CD4 | BD Horizon^TM^ | Cat # 562281/562316 |
| PE-Cy^TM^7 Mouse Anti-Human CD8 | BD Pharmingen^TM^ | Cat # 557872 |
| PerCP-Cy^TM^5.5 Mouse Anti-Human CD8 | BD Pharmingen^TM^ | Cat # 560662 |
| FITC Mouse Anti-Human CD45 | BD Pharmingen^TM^ | Cat # 560989 |
| PE Mouse Anti-Human CD25 | BD Pharmingen^TM^ | Cat # 560976/561865 |
| Alexa Fluor647 Mouse Anti-Human CD127 | BD Pharmingen^TM^ | Cat # 558598 |
| PE Rat Anti-Human CCR7 (CD197) | BD Pharmingen^TM^ | Cat # 552176/561008 |
| APC Mouse Anti-Human CD45RA | BD Pharmingen^TM^ | Cat # 555745 |

**Enzyme-linked immunosorbent assay (ELISA)**

Overall, 32 patient ascites samples from the SE-iFISH cohort (Cohort-1) were used, in which the C1q, C2, C3, and C4 complement factors were measured using ELISA kits. All samples were derived from the Peking University Cancer Hospital and stored at −80 ℃. All ELISA kits were used according to the manufacturer’s instructions. The samples were run in duplicates, and the values were presented as means±standard deviations (SDs) of three independent experiments and analyzed individually.

**Reagent kits were used in ELISA**

| REAGENT KITS | SOURCE | | IDENTIFIER | |
| --- | --- | --- | --- | --- |
| The Human complement 1q (C1q) ELISA kit | CUSABIO | Cat # CSB-E10118h | |  |
| Human complement C2 (C2) ELISA kit | CUSABIO | Cat # CSB-EL003658HU | |  |
| Human complement C3 (C3) ELISA kit | CUSABIO | Cat # CSB-E08665h | |  |
| Human complement C4 (C4) ELISA kit | CUSABIO | Cat # CSB-E08705h | |  |
| BCA Protein Assay Kit | TIANGEN | Cat # PA115-01 | |  |

**Western blot assay**

Total protein was extracted using a RIPA Lysis Buffer, and the protein concentration was measured using the BCA Protein Assay Kit (Beyotime Biotechnology, Jiangsu, China) according to the manufacturer’s instructions. For western blot analysis, 30–50 μg protein samples were separated on a 10% SDS PAGE gel and then transferred to polyvinylidene fluoride (PVDF) membranes. The blots were incubated overnight in a blocking buffer (5% skim milk in Tris-HCl salt solution buffer with 0.1% Tween 20) with primary antibodies at 4 °C. After being washed three times with Tris-HCl salt solution buffer with 0.1% Tween 20, the blots were probed with a horseradish peroxidase-conjugated secondary antibody at 20°C–25°C for 1 h and developed with the Super-signal West Pico or Dura (Thermo Fisher Scientific, Waltham, MA, USA).

**Antibodies used in western blot assay**

| ANTIBODIES | SOURCE | IDENTIFIER |
| --- | --- | --- |
| Rabbit monoclonal anti-GAPDH antibody | Cell Signaling Technology | Cat # 5174S |
| Rabbit monoclonal anti-PD-L1 antibody | Cell Signaling Technology | Cat # 13684S |
| Rabbit monoclonal anti-NECTIN-2 antibody | Cell Signaling Technology | Cat # 95333T |
| Anti-Rabbit immunoglobulin-G,  and HRP-linked antibody | Cell Signaling Technology | Cat # 7074s |
| HRP-conjugated Streptavidin | Proteintech | SA00001-0 |

**Software and algorithms**

| SOFTWARE and ALGORITHMS | SOURCE | IDENTIFIER |
| --- | --- | --- |

| Metafer CTC 3D scanning and image analysis system | MetaSystems | https://metasystems-international.com/ |
| --- | --- | --- |
| CeleScope v1.3.0 | Singleron | https://github.com/singleron-RD/CeleScope |
| FastQC v0.11.4 | Butler et al., 2018 | https://www.bioinformatics.babraham.ac.uk/projects/fastqc/ |
| Fastp | Chen et al., 2018 | https://github.com/OpenGene/fastp |
| STAR v2.5.3a | Chen et al., 2018 | https://github.com/alexdobin/STAR |
| Counts v1.6.2 | Liao et al., 2014 | https://subread.sourceforge.net/featureCounts.html |
| Scanpy v1.8.2 | Wolf et al., 2018 | https://scanpy.readthedocs.io/en/stable/ |
| Seurat v3.1.2 | Satija et al., 2015 | https://satijalab.org/seurat/ |
| SynEcoSys database | Singleron | https://data.humancellatlas.org/analyze/portals/synecosys |
| InferCNV package | Kumar et al., 2020 | https://jlaffy.github.io/infercna |
| Monocle 2 | Qiu et al., 2017 | <https://github.Scylardor/Monocle2> |
| DDR Tree | Shi et al., 2020 | https://github.com/cole-trapnel-lab/DDRTree |
| CellPhoneDB v2.1.0 | Efremova et al., 2020 | https://github.com/Teichlab/cellphonedb |
| Circlize v0.4.10 R package | Gu et al., 2014 | https://jokergoo.github.io/2020/06/14/changes-in-circlize-0.4.10/ |
| Amersham Imager 680 | Cytiva | https://www.cytivalifesciences.com.cn/zh/cn/shop/protein-analysis/molecular-imaging-for-proteins/imaging-system/amersham-imagequant-800-system-p-11546 |
| FlowJo V | FlowJo LLC. | https://www.flowjo.com/ |
| SPSS 21.0 | IBM Corp | https://www.ibm.com/cn-zh/spss |
| GraphPad Prism V7 | GraphPad Software | https://www.graphpad.com/scientific-software/prism/ |

| Origin 2022 | OriginLab | https://www.originlab.com/2022 |
| --- | --- | --- |
